# Overlap between COPD genetic association results and transcriptional quantitative trait loci

**DOI:** 10.1016/j.xhgg.2025.100493

**Published:** 2025-08-11

**Authors:** Aabida Saferali, Wonji Kim, Robert P. Chase, NHLBI TransOmics in Precision Medicine (TOPMed), Christopher Vollmers, Edwin K. Silverman, Michael H. Cho, Peter J. Castaldi, Craig P. Hersh

**Affiliations:** 1 Channing Division of Network Medicine, Brigham and Women’s Hospital, Boston, MA, USA; 2 Department of Biomolecular Engineering, Cellular, Cellular, and Developmental Biology, University of California, Santa Cruz, Santa Cruz, CA, USA; 3 Division of Pulmonary and Critical Care Medicine, Brigham and Women’s Hospital, Boston, MA, USA; 4 Division of General Medicine and Primary Care, Brigham and Women’s Hospital, Boston, MA, USA

**Keywords:** splicing, quantitative trait loci, chronic obstructive pulmonary disease, genetic colocalization

## Abstract

Genome-wide association studies (GWASs) have identified multiple genetic loci associated with chronic obstructive pulmonary disease (COPD). Here, we identify SNPs that are associated with alternative splicing (splicing quantitative trait loci [sQTLs]) and gene expression (expression QTLs [eQTLs]) to identify functions for COPD-associated genetic variants. RNA sequencing on whole blood from 3,743 subjects in the COPDGene Study and from lung tissue of 1,241 subjects from the Lung Tissue Research Consortium (LTRC) was analyzed. Associations between all SNPs within 1,000 kb of a gene (*cis*-) and splice and gene expression quantifications were tested using tensorQTL. We assessed colocalization with COPD-associated SNPs from a published GWAS. After adjustment for multiple statistical testing, we identified 28,110 splice sites corresponding to 3,889 unique genes that were significantly associated with genotype in COPDGene whole blood and 58,258 splice sites corresponding to 10,307 unique genes associated with genotype in LTRC lung tissue. To determine what proportion of COPD-associated SNPs were associated with transcriptional splicing, we performed colocalization analysis between COPD GWAS and sQTL data and found that 38 genomic windows, corresponding to 33 COPD GWAS loci, had evidence of colocalization between QTLs and COPD. The top five colocalizations between COPD and lung sQTLs include *Nephronectin* (*NPNT*), *F box protein 38* (*FBXO38*), *Hedgehog interacting protein* (*HHIP*), *Netrin 4* (*NTN4*), and *Betacellulin* (*BTC*). Overall, a total of 38 COPD GWAS loci contain evidence of sQTLs, suggesting that analysis of sQTLs in whole blood and lung tissue can provide insights into disease mechanisms.

## Teaser

While it is known that chronic obstructive pulmonary disease (COPD) is caused in part by genetic factors, few studies have identified specific causative genes. Here, we found that genetic variants that affect RNA splicing in blood and lung tissue can explain many COPD disease associations.

## Introduction

Chronic obstructive pulmonary disease (COPD) is a complex disease characterized by irreversible airflow obstruction on lung function testing. The leading environmental risk factor for COPD is cigarette smoking; however, genetic factors have also been shown to play a role in disease susceptibility.^1^^,^^2^^,^^3^^,^^4^ Genome-wide association studies (GWASs) have been used to identify genetic variants associated with COPD and lung function.^5^^,^^6^^,^^7^^,^^8^ However, as for most complex trait GWAS associations, the causal mechanisms are currently unknown. While it has been found that expression quantitative trait loci (eQTL) are enriched among GWAS loci, a large proportion of disease heritability remains unexplained by eQTLs.^9^ Previous work has shown that splicing QTLs (sQTLs), in which genetic variants affect alternative splicing, can account for at least as many GWAS loci as eQTLs.^10^

A recent GWAS for COPD, including 35,735 affected individuals and 222,076 control subjects from the UK Biobank and the International COPD Genetics Consortium, identified 82 loci associated with COPD with genome-wide significance.^5^ Using S-PrediXcan,^11^ the authors discovered that 49 GWAS loci had evidence for genetically regulated expression associated with COPD using data from the Lung-eQTL consortium.^5^ As S-PrediXcan is also influenced by linkage disequilibrium (LD), most of the COPD GWAS loci are likely not explained using existing eQTLs.

Genomic loci identified as being eQTLs may also be sQTLs, as splicing is a common mechanism to alter total gene expression levels. In our previous work, we generated sQTLs in 376 subjects from the COPDGene study and found that these data could explain seven COPD GWAS associations, including the identification of *FBXO38* as a novel COPD susceptibility gene at 5q32.^10^ Here, we expand upon our findings by developing a large database of eQTLs and sQTLs in RNA from lung tissue from 1,241 subjects and in RNA from blood from 3,743 subjects followed by colocalization analysis with COPD GWAS results.

## Material and methods

### Study population

This study included 3,713 non-Hispanic White and African American subjects from the COPDGene study and 1,241 subjects from the Lung Tissue Research Consortium (LTRC) (Table 1). COPDGene enrolled individuals between the ages of 45 and 80 years with a minimum of 10 pack-years of lifetime smoking history from 21 centers across the United States.^12^ These subjects returned for a second study visit 5 years after the initial visit, at which time they completed additional questionnaires, pre- and post-bronchodilator spirometry, and computed tomography (CT) of the chest and provided blood for complete blood counts (CBCs) and RNA sequencing (RNA-seq). Samples were collected as part of the LTRC from individuals who were undergoing clinically indicated thoracic surgery procedures using a standardized protocol described in the original study design, which included pulmonary function testing, questionnaires, and chest CT. The COPDGene and LTRC studies have been approved by Mass General Brigham IRB (Protocol # 2007P000554 and 2018P000186).^13^

**Table 1:** Clinical characteristics of LTRC and COPDGene study individuals included in the analysis

	LTRC (*n* = 1241)	COPDGene (*n* = 3734)
Gender, male (%)	52.4	51.1
Age, mean (SD)	63.3 (10.6)	59.9 (8.7)
Race, *n* (%)		
White	1,118 (90.1)	2,702 (72.4)
Asian	4 (0.3)	–
Black	81 (6.5)	1,032 (27.6)
Hispanic	25 (2.0)	–
Other	13 (1.0)	–
Current smokers, *n* (%)^a^	73 (5.9)	1,766 (47.3)
Pack-years smoked, mean (SD)	31.8 (±33.2)	42.7 (±24.0)

a Smoking data are missing for a subset of LTRC subjects.

### RNA-seq, alignment, and count generation

The protocols for RNA-seq data generation and processing for COPDGene and LTRC have been previously described.^13^^,^^14^ Briefly, for LTRC, mRNA sequencing (RNA-seq) was performed through the NHLBI TOPMed program at the University of Washington. Poly(A) selection and cDNA synthesis were performed using the TruSeq Stranded mRNA kit (Illumina, San Diego, CA), and sequencing was performed on the NovaSeq6000 instrument. Sequences were aligned to GRCh38 using STAR (v.2.6.1d) with the GENCODE release 29 reference. Gene-level expression quantification was performed using RSEM (v.1.3.1). For COPDGene, globin reduction, ribosomal RNA depletion, and cDNA library prep were performed on total RNA from whole blood using the TruSeq Stranded Total RNA with Ribo-Zero Globin kit (Illumina), and sequencing was performed on Illumina platforms. Sequences were aligned to GRCh38 using STAR 2-pass alignment (v.2.5.2b). Gene-level expression quantification was performed using Salmon (v.1.3.0) for pseudoalignment to the GENCODE release 37 transcriptome, followed by summarization of isoform-level counts to gene-level counts using tximport (v.1.8.5).^15^^,^^16^ Quantification of splicing ratios was performed in COPDGene and LTRC using LeafCutter with default parameters.^17^ Intron ratios were calculated by determining how many reads support a given exon-intron junction in relation to the number of reads in that region. LTRC RNA-seq data are available through TOPMed (https://topmed.nhlbi.nih.gov). COPDGene data are available on dbGaP with accession numbers phs000179 and phs000765.

### Whole-genome sequencing

All samples were sequenced through the TOPMed program. This analysis uses Freeze 8 data. LTRC genotyping data are available on dbGaP with accession number phs001662.v1.p1.

### eQTL and sQTL analysis

Gene expression counts were filtered to include only genes with at least 1 count per million in at least 20% of subjects, and the remaining counts were TMM normalized.^18^ LeafCutter ratios were filtered to remove introns detected in less than 40% of individuals and introns with a standard deviation of less than 0.005 across subjects, and the remaining ratios were scale normalized (i.e., mean centered and divided by the standard error). TensorQTL^19^ was used to test for association between genotypes of all SNPs within 1,000 kb of the gene boundary (*cis*-) and quantifications of splicing or gene expression using linear models, adjusting for gender, RNA-seq library preparation batch, principal components (PCs) of splicing (10 PCs) or gene expression data (170 PCs for COPDGene and 190 PCs for LTRC), and PCs of genetic ancestry (10 PCs). The calculation of PCs of genetic ancestry has been previously described.^20^ A total of 8,792,206 variants (bi-allelic SNPs with a mean-allele frequency [MAF] > 0.01) were tested for association with 58,258 splice sites (corresponding to 10,615 unique genes) and expression of 16,264 genes. Results were annotated using ANNOVAR^21^ with annotations derived from dbSNP build 150. Genotype Tissue Expression Project (GTEx) v.8 significant results^22^ were obtained for all tissues from the GTEx Portal (https://www.gtexportal.org/home/datasets), and complete lung sQTL results were obtained from the Anvil GTEx Terra workspace.

### Colocalization analysis

Published GWAS data for COPD case-control status^5^ were used for this analysis. Testing windows were generated by identifying all GWAS variants with *p* < 1E^−5^ and calculating non-overlapping windows of 1 Mb on either side of each SNP. Only windows containing sQTLs or eQTLs with a false discovery rate (FDR) < 0.05 were tested. The 82 genome-wide significant loci and 89 testing windows are shown in Table S1. For each window, Bayesian colocalization tests were performed using the Moloc R package^23^ to quantify the probability that the GWAS and sQTL or eQTL associations were due to a shared causal variant. Windows with a colocalization posterior probability (CPP) of greater than 0.8 were reported. Fine mapping was performed on QTL results from selected regions of interest using SuSieR v.12.35, with in-sample LD.^24^

### Long-read RNA-seq analysis in human lung samples from the LTRC

We conducted targeted Oxford Nanopore Technologies (ONT) long-read sequencing on RNA from 170 human lung samples from the LTRC on genes selected from colocalization analysis. The enrichment and library generation procedures are described in detail in the supplemental methods. The final library was loaded onto a PromethION R10.4 flow cell and run at 400 bp/s. Approximately once per day, flow cells were flushed and treated with DNase I and then loaded with an additional library to increase sequencing throughput. Resulting raw reads were base called using the SUP model of guppy (v.6) and consensus called and demultiplexed using C3POa (v.2.3). R2C2 reads were analyzed to identify and quantify isoforms using v.3.5 of Mandalorion (https://github.com/rvolden/Mandalorion-Episode-III). Mandalorian was run twice to identify high-abundance isoforms (>10% of total isoform expression; -O 0,40,0,40 -r 0.1 -i 1 -w 1 -n 2 -R 5) and high- and low-abundance isoforms (-O 0,40,0,40 -r 0.01 -i 1 -w 1 -n 2 -R 5).

## Results

### Quantification of gene expression and RNA splicing in COPDGene blood and LTRC lung

Using RNA-seq data from LTRC lung tissue (*n* = 1,241) and COPDGene whole blood (*n* = 3,743), we identified splice sites using LeafCutter,^17^ which identifies and quantifies intron exclusion. After filtering out splice sites with low usage, we identified a total of 223,153 splice sites corresponding to 15,120 genes in LTRC and 160,655 splice sites corresponding to 12,096 genes in COPDGene (Table 2). In both LTRC and COPDGene, the majority (50.1% and 51.0%, respectively) of identified splice sites were annotated in GENCODE, followed by cryptic 3′, cryptic 5′, cryptic unanchored splice sites (meaning both splice donor and acceptor were unannotated), and novel annotated pairs (Table 3). The gene expression of 16,264 genes in LTRC and 15,507 genes in COPDGene met expression thresholds (Table 2).

**Table 2:** Summary of eQTLs and sQTLs tested

	COPDGene		LTRC	
	Genes	Splice sites	Genes	Splice sites
Before filtering	60,232	237,155	58,962	306,475
After filtering	15,507	160,655	16,266	223,153
Genes tested	15,507	12,096	16,266	15,120
SNPs tested	11,869,333	11,869,333	8,792,206	8,792,206
Significant QTLs^a^	15,279	63,946	12,225	58,258

a The number of genes or splice sites significantly associated with at least one SNP with an FDR < 0.05.

**Table 3:** Annotations of LeafCutter splice sites identified in COPDGene and LTRC

	Frequency in LTRC	Percentage in LTRC	Frequency in COPDGene	Percentage in COPDGene
Annotated	113,562	50.9	81,111	50.5
Cryptic 5′	30,166	13.5	21,232	13.2
Cryptic 3′	32,258	14.5	24,047	15.0
Cryptic unanchored	8,504	3.8	5,788	3.6
Novel annotated pair	17,991	8.1	13,293	8.3
Unknown strand	20,651	9.2	15,166	9.4

Annotated: both 5′ and 3′ splice sites have been previously annotated. Cryptic 5′: the 5′ splice site is not annotated, but the 3′ splice site is annotated. Cryptic 3′: the 3′ splice site is not annotated, but the 5′ splice site is annotated. Cryptic unanchored: neither splice site has been previously annotated. Novel annotated pair: both 5′ and 3′ splice sites have been individually annotated, but the combination has not been annotated as a junction. Unknown strand: it is not possible to determine the directionality of the splice sites based on the RNA-seq read.

### Identification of eQTL and sQTLs in human lung tissue and whole blood

We next tested for associations between genotype and gene expression or splicing to identify eQTLs and sQTLs in lung and blood. In LTRC lung tissue, we identified 58,258 splice sites (corresponding to 10,615 genes) associated with at least one SNP with *q* < 0.05; in COPDGene blood, 63,946 splice sites (corresponding to 9,433 genes) were associated with at least one SNP (Table 2). In addition, we identified 12,225 genes associated with at least one SNP (eQTLs) in LTRC and 15,279 eQTL genes in COPDGene. We next investigated what proportion of the QTLs found in our data were previously identified in GTEx lung (*n* = 515) and whole blood (*n* = 670). We found that out of 18,688 splice clusters that were significantly associated with at least one SNP in LTRC lung, 3,148 had sQTLs in GTEx lung, and of 18,294 COPDGene sQTL clusters, 1,848 were significant in GTEx blood. For eQTLs, 5,494 out of 12,225 LTRC eGenes were also eGenes in GTEx lung, and 5,753 out of 15,279 eGenes in COPDGene were also eGenes in GTEx blood (Figure S1).

**Figure 1:**
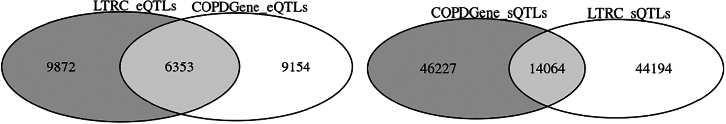
Overlap between LTRC lung and COPDGene blood eQTLs and sQTLs that were significant at an FDR < 0.05

We found that 14,946 sQTL splice-site-SNP pairs (13%) were found in both COPDGene blood and LTRC lung, while 6,349 eQTL gene-SNP pairs were shared between both tissues (25%) (Figure 1). In addition, we found that 5,787 sQTL gene-SNP pairs overlapped with eQTL gene-SNP pairs in LTRC (47%), while 7,455 sQTL gene-SNP pairs overlapped with eQTL gene-SNP pairs in COPDGene (49%).

### Functional annotation of sQTLs and eQTLs

Next, we categorized eQTLs and sQTLs-SNPs on the basis of their location relative to the gene with which they were associated (Table 4). The genomic distribution of eQTLs and sQTLs was similar, with the largest proportion of variants located upstream of the gene region in both COPDGene (32.1% and 30.1%, respectively) and LTRC (31.8% and 29.4%, respectively). The next most frequent SNPs were intronic, downstream of a gene, intergenic, and 3′ UTR variants. Only a small percentage of lead sQTL-SNPs directly modified a splice site. There was no difference in variant position in either eQTL vs. sQTL or COPDGene blood vs. LTRC lung.

**Table 4:** Annotation of QTL variants in relation to the gene body

Annotation	COPDGene		LTRC	
	eQTLs (%)	sQTLs (%)	eQTLs (%)	sQTLs (%)
Upstream gene variant	32.1	30.1	31.8	29.4
Intron variant	24.4	28.8	25.2	25.8
Downstream gene variant	14.5	14.9	14.9	15.8
Intergenic region	11.9	9.7	10.6	11.4
3′ UTR variant	5.0	3.4	5.7	3.3
5′ UTR variant	3.1	2.4	3.6	2.2
Missense variant	2.1	2.5	2.2	3.3
Synonymous variant	1.9	2.3	1.8	3.2
Other	4.9	5.8	2.8	4.2

### Colocalization of QTLs with COPD case-control GWAS data

We next sought to identify eQTLs and sQTLs that contribute to COPD risk by performing genetic colocalization between the QTL data and COPD case-control GWAS data. The 82 previously identified COPD GWAS loci^5^ correspond to 89 windows of 2 Mb in width (Table S1). We also included colocalization results for 3′ UTR alternative polyadenylation QTLs (apaQTLs) from our previous study.^25^ We identified 38 windows (corresponding to 32 of the 82 GWAS loci) with a colocalization posterior probability of association (PPA) > 0.8 with either eQTL, sQTL, or apaQTL data and GWAS *p* < 5.0E−8 (Table 5). We found that for 19 out of the 82 COPD loci, the strongest colocalization (largest PPA) with GWAS data was in LTRC, and for 19 loci, the strongest colocalization was in COPDGene. In LTRC, the largest number of GWAS loci colocalized with sQTLs (9 loci), followed by eQTLs (7 loci) and then apaQTLs (3 loci). In COPDGene, the majority of colocalizations were with sQTLs (15 loci), with only one 1 locus colocalizing most strongly with eQTLs and 3 with apaQTLs. We compared the colocalization findings with the target genes identified from the original GWAS analysis to determine whether the QTLs identified additional targets from previous analyses. We found that for 7 loci, all genes identified in the current QTL analysis were previously identified, and for 26 loci, one or more new targets were found (Table S2). For further characterization, we focused on sQTL colocalizations in LTRC lung, with the top five colocalizations (by highest PPA) being *Nephronectin* (*NPNT)*, *F box protein 38* (*FBXO38*), *Hedgehog interacting protein* (*HHIP*), *Netrin 4* (*NTN4*), and *Betacellulin* (*BTC*). We have previously published on NPNT colocalizations in the lung,^26^ and for HHIP, significant evidence suggests that the mechanism underlying the association is an eQTL effect.^27^ *NTN4* appears to be a promoter-usage eQTL instead of an sQTL. Therefore, we highlight the results for *FBXO38* and *BTC* below. All colocalization results are available online at https://copd-moloc.bwh.harvard.edu/.

**Table 5:** Summary of colocalization analysis

Best colocalized SNP^a^	PPA^b^	Lead QTL					GWAS	
		Gene^b^	QTL type	Splice site	*p* value^d^	Effect^c^	*p* value	Effect^c^
chr4:144567946A>G	0.973	*HHIP*	LTRC_sQTL	chr4:144734889–144737129	0.00610	0.104	4.09E−59	16.136
chr4:105897896G>A	0.999	*NPNT*	LTRC_sQTL	chr4:105927428–105931515	8.12E−10	0.114	3.04E−46	14.331
chr5:148475407C>T	0.973	*FBXO38*	LTRC_sQTL	chr5:148414306–148415928	0.00333	−0.195	2.58E−33	−12.010
chr15:71329185G>A	0.992	*THSD4*	LTRC_eQTL	ENSG00000187720.14	0.154	4.653	1.58E−32	11.852
chr16:75439564G>A	0.933	*TMEM170A*	COPDGene_sQTL	chr16:75451839–75464232	7.63E−08	0.187	1.26E−20	9.277
chr6:30814428G>C	0.987	*IER3*	COPDGene_sQTL	chr6:30744196–30744279	0.000119	−0.176	1.41E−20	9.295
chr4:88948181T>C	0.832	*PKD2*	COPDGene_sQTL	chr4:88019571–88035071	3.76E−08	−0.518	4.23E−18	−8.722
chr6:32660630T>C	0.997	*HLA-DRB5*	COPDGene_sQTL	chr6:32520345–32555661	6.09E−15	−0.275	5.56E−18	−8.661
chr5:157505976A>T	0.968	*ADAM19*	LTRC_eQTL	ENSG00000135074.15	8.62E−10	2.468	1.22E−16	−8.324
chr3:128242335T>A	0.935	*EEFSEC*	COPDGene_sQTL	chr3:128195329–128246836	0.0014	0.032	3.53E−15	7.897
chr6:29639324T>C	0.997	*HLA-E*	COPDGene_sQTL	chr6:30490515–30491137	4.82E−301	−0.890	4.35E−15	−7.864
chr6:27420975T>C	0.976	*ZKSCAN4*	COPDGene_sQTL	chr6:28249834–28251867	5.35E−20	0.308	3.58E−14	−7.573
chr6:28651576A>G	0.996	*GABBR1*	COPDGene_eQTL	ENSG00000204681.11	3.96E−17	−2.034	3.82E−14	−7.556
chr6:26409662G>C	0.905	*BTN2A1*	LTRC_sQTL	chr6:26459828–26463244	1.79E−10	−0.486	1.03E−13	7.449
chr6:32775967G>A	0.982	*HLA-DRB5*	COPDGene_sQTL	chr6:32481801–32525584	8.02E−09	−0.222	3.76E−12	6.955
chr2:238965524G>A	0.941	*TWIST2*	LTRC_eQTL	ENSG00000233608.4	0.0483	0.269	2.17E−11	−6.699
chr3:25496178G>A	0.990	*RARB*	LTRC_eQTL	ENSG00000077092.19	2.60E−06	0.925	5.84E−11	−6.570
chr2:9145396G>A	0.956	*LINC00299*	COPDGene_sQTL	chr2:8299793–8312726	0.0479	−0.115	2.41E−10	6.343
chr1:16979534C>A	0.990	*CROCC*	COPDGene_sQTL	chr1:16922798–16924325	0.000528	−0.121	6.55E−10	6.192
chr7:100032719C>T	0.994	*ZSCAN21*	COPDGene_sQTL	chr7:100049841–100051478	6.28E−05	−0.153	7.26E−10	−6.175
chr19:45790878G>A	0.998	*DMWD*	LTRC_APAQTL	ENST00000597053.1	0.00118	−0.044	1.67E−09	−6.009
chr1:39582337G>A	0.933	*PPIEL*	COPDGene_sQTL	chr1:39548951–39554349	0.0176	−0.128	1.98E−09	6.009
chr12:95843792T>C	0.972	*NTN4*	LTRC_sQTL	chr12:95713338–95717545	6.56E−09	−0.400	2.64E−09	−5.935
chr4:74748514C>T	0.951	*BTC*	LTRC_sQTL	chr4:74748149–74750573	0.00187	−0.238	3.45E−09	−5.903
chr1:111195294T>C	0.850	*DENND2D*	COPDGene_sQTL	chr1:111194726–111195668	0.00843	−0.556	3.59E−09	−5.892
chr14:92649065G>C	0.963	*RIN3*	LTRC_APAQTL	ENST00000553992.1	0.00115	−0.025	6.69E−09	5.795
chr1:239689643G>C	0.968	*CHRM3-AS2*	COPDGene_APAQTL	ENST00000593855	2.77E−48	−0.279	9.61E−09	5.755
chr11:13145018T>C	0.941	*RASSF10*	LTRC_eQTL	ENSG00000189431.7	4.27E−09	0.250	1.18E−08	5.717
chr5:151215512A>G	0.994	*GM2A*	LTRC_APAQTL	ENST00000523004.1	9.43E−100	0.011	1.40E−08	−5.695
chr7:2830820T>G	0.873	*GNA12*	LTRC_sQTL	chr7:2733501–2762829	0.00185	0.156	1.74E−08	5.648
chr17:38730575G>A	0.990	*CISD3*	LTRC_eQTL	ENSG00000277972.1	8.70E−11	−2.872	1.90E−08	5.620
chr3:29430921G>C	0.985	*RBMS3*	LTRC_eQTL	ENSG00000144642.21	1.02E−11	−2.128	2.04E−08	5.636
chr15:49578340A>G	0.943	*FAM227B*	LTRC_sQTL	chr15:49589745–49606156	0.0146	−0.151	2.82E−08	5.564
chr16:58063696G>C	0.890	*MMP15*	LTRC_sQTL	chr16:58040698–58041617	0.0122	0.274	4.26E−08	5.500
chr10:80458350G>C	0.995	*TSPAN14*	COPDGene_sQTL	chr10:80459507–80463088	3.64E−07	0.132	4.30E−08	−5.465
chr1:45543641G>C	0.959	*TESK2*	COPDGene_APAQTL	ENST00000486676	0.00967	−0.032	4.67E−08	−5.485
chr2:42206107C>T	0.980	*COX7A2L*	COPDGene_APAQTL	ENST00000463055	N/A	N/A	4.85E−08	−5.446
chr17:30129740C>T	0.865	*NSRP1*	COPDGene_sQTL	chr17:30118173–30156218	0.000150	0.116	4.86E−08	5.480

a SNP: chromosome, position, reference, and effect allele, where position is build 38.

b PPA: posterior probability of association from Moloc software. For some loci, there were multiple colocalizations between QTLs and GWAS data; the results for the association with the highest PPA are reported here.

c The direction of the effect size is based on the second allele listed in the variant ID.

d *p* value is for the QTL shown in the “QTL type” column.

### Characterization of sQTL for FBXO38

**Figure 2:**
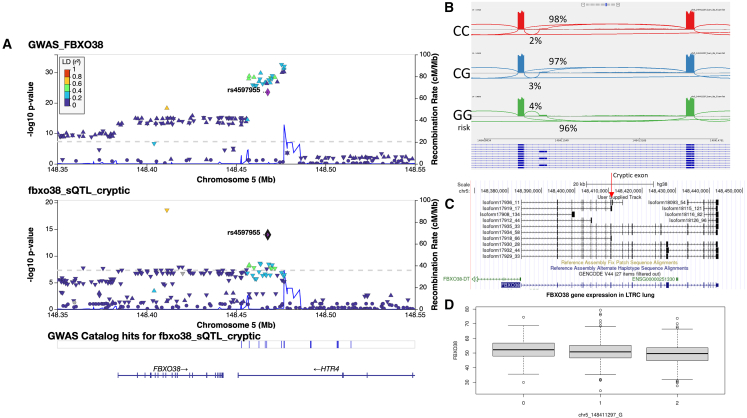
Replication of FBXO38 sQTL findings in lung tissue (A) Locus association plot for COPD GWAS and *FBXO38* sQTLs. The lead SNP associated with FBXO38 splicing, rs7730971, is highlighted in purple and used as the LD reference. (B) IGV sashimi plot showing the region spanning chr5:148409934–148414781 from lung tissue RNA-seq from 191 subjects from each genotype of rs7730971. (C) Long-read sequencing data showing all FBXO38 isoforms representing at least 1% of total FBXO38 expression. The cryptic exon is identified with a red arrow. (D) Boxplot of total gene expression values for *FBXO38*.

We first sought to replicate the association we previously characterized in 365 subjects from COPDGene between the rs7730971 (GRCh38 chr5:148411297C>G) variant and *FBXO38* splicing in whole blood, which colocalized with COPD GWAS findings.^10^ Here, we identified two splicing clusters (i.e., a group of splice junctions with shared start or stop positions^17^) in lung tissue in *FBXO38*, which were associated with COPD-related variants. First, we confirmed our previous finding that rs7730971 was significantly associated with the inclusion of a 158 bp cryptic exon located between exons 9 and 10 (Figure 2). While significant colocalization was not detected using Moloc, we previously found colocalization in this locus using eCaviar.^28^ The G allele, which is associated with increased risk of COPD, is also associated with increased inclusion of the cryptic exon (Figure 2B). SpliceAI^29^ indicates that rs7730971-G is predicted to slightly increase the splice acceptor strength of a splice site 217 bp upstream of the variant, which corresponds to the 5′ splice acceptor of the cryptic exon, confirming that this is the likely causal variant. Using long-read sequencing, we identified one isoform meeting expression thresholds, which includes the 158 bp cryptic exon (Figure 2C). This transcript includes a premature stop codon in the cryptic exon, and because this stop is more than 50 bases from the transcriptional stop, it would likely be subject to nonsense-mediated decay.^30^ This isoform is more abundant in the GG genotype compared to AA (5 reads vs. 1). Supporting the finding that the transcript containing the cryptic exon is subject to nonsense-mediated decay, we found that rs7730971 is also an eQTL for *FBXO38*, with the G allele associated with decreased expression (Figure 2D). We additionally identified a new colocalization between genetic variants in the *FBXO38/HTR4* region and inclusion of an exon at GRCh38 chr5:148415241–148415387 (supplemental information).

### Colocalization analysis of a genetic signal at BTC

**Figure 3:**
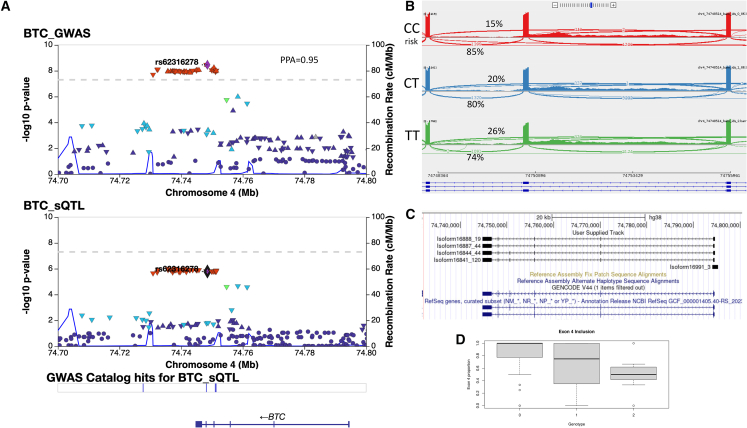
sQTLs for BTC colocalize with GWAS data for COPD (A) Locus association plot for COPD GWAS and *BTC* sQTL. The lead colocalized SNP, rs62316278, is highlighted in purple and used as the LD reference. (B) IGV sashimi plot showing the region spanning chr4:74748364–74755961 from lung tissue RNA-seq from 86 subjects from each genotype of rs62316278. (C) Long-read sequencing data showing all BTC isoforms representing at least 10% of total BTC expression. (D) Proportion of BTC expression of isoforms containing exon 4 by copies of rs62316278-T.

Another significant colocalization between sQTLs and COPD GWAS findings was identified in LTRC lung tissue at the *BTC* locus, where we found evidence of a shared genetic signal between variants associated with alternative inclusion of exon 4 of *BTC*, and COPD risk GWAS data (PPA = 0.95) (Figure 3A). The lead colocalized variant was rs62316278 (GRCh38 chr4:74748514C>T), and the COPD risk allele (C) is associated with increased inclusion of exon 4 (Figure 3B). This variant is within the 95% credible set for sQTL data using SuSie fine mapping, along with 42 additional variants. Of the variants in the 95% credible set, we found using SpliceAI that rs11938093-T (GRCh38 chr4:74750631A>T) is associated with the gain of a splice donor 58 bp upstream of the SNP, corresponding to the splice donor of exon 4, as well as the gain of a splice acceptor 88 bp downstream of the SNP, corresponding to the splice acceptor of exon 4. Long-read sequencing identified four high-abundance isoforms for *BTC* (representing at least 10% of BTC expression each) (Figure 3C), and the proportion of isoforms containing exon 4 was higher in CC vs. TT subjects (84% vs. 48%, *p* = 0.0003). These isoforms correspond to GenBank: NM_001729 (BTC-201, ENST00000395743.8) and NM_001316963 (not included in ENSEMBL). GenBank: NM_001729 (including exon 4) is the primary version of BTC and codes for a 178 amino acid protein, while GenBank: NM_001316963 codes for 129 amino acids.

## Discussion

In this study, we build upon our previous work characterizing COPD-associated sQTLs in blood RNA-seq data from COPDGene by generating a large dataset of eQTLs and sQTLs in human blood and lung tissue and identifying gene expression and splicing events, and we identify a substantial number of QTLs that suggest a functional mechanism for COPD GWAS loci. We found that approximately 50% of splice sites identified were not currently annotated, indicating the vast amount of currently uncharacterized splicing variability present in the transcriptome. Among the 223,128 splice sites identified in LTRC lung tissue and the 160,658 splice sites identified in COPDGene blood, we found that 58,258 (26%) and 60,291 (38%) splice sites, respectively, were associated with at least one variant within 1 Mb (FDR < 5%). In addition, of the 16,266 genes expressed in LTRC and 15,507 genes expressed in COPDGene, 12,225 (75%) and 15,279 (99%) of genes, respectively, were associated with at least one SNP. The majority of eQTL and sQTL SNPs were located upstream of the gene body or in intronic regions, suggesting that many sQTLs function through long-range or indirect effects, as opposed to modifying splice donors or acceptors directly. Alternatively, the identified SNPs may be tagging another SNP in LD, which more directly modifies a splice site. We identified 38 loci (corresponding to 33 of the original 82 GWAS loci) with significant colocalization with either eQTL, sQTL, or apaQTL data, and of these, 9 loci colocalized most strongly with LTRC sQTLs and 15 with COPDGene sQTLs. We confirmed our previous sQTL findings in the *FBXO38/HTR4* region and identified *BTC* as additional target with strong COPD colocalization.

Here, we validated in lung tissue our previous findings from blood that rs7730971 is associated with splicing of a cryptic exon in *FBXO38*. Open reading frame analysis of the full length transcript sequence indicates that the cryptic exon contains a premature stop codon, and therefore, this transcript is likely subject to nonsense-mediated decay. We also found that rs7730971 is associated with gene expression of *FBXO38*, with decreased expression with the G allele, which is also the variant associated with a bioinformatically predicted increase in nonsense-mediated decay. Therefore, the likely mechanism underlying the eQTL association is degradation of *FBXO38* as result of increased inclusion of a cryptic exon, which results in a transcript with an early stop codon. This is an example of a mechanism by which an eQTL can be mediated through an sQTL. While the difference in inclusion of cryptic exon is small, 2% vs. 4% inclusion in the CC vs. GG genotype, this measurement is likely skewed, as the transcript that contains the cryptic exon would be degraded, resulting in decreased quantification of exon inclusion. In other words, the inclusion of the exon in the GG genotype is likely inaccurate due to the degradation of the isoform that contains the exon. Future directions include inhibition of nonsense mediation followed by RNA-seq to quantify the true levels of exon inclusion.

The allele associated with decreased expression of *FBXO38* is associated with increased COPD risk, suggesting that *FBXO38* plays a protective role against COPD. We found an additional colocalization between rs10037493, the most significant COPD GWAS SNP in the locus, and an additional *FBXO38* exon (shown in the supplemental results). The long-read sequences containing this exon correspond to two predicted isoforms, each encoding shorter proteins than the most abundant isoform. The COPD risk allele is associated with the shortened isoforms, indicating that the full-length *FBXO38* isoform is protective. These shortened isoforms lack the F box domain, which is a component of all members of F box proteins, including FBXO38. F box proteins are a component of the ubiquitin ligase complex and also function as transcription factors.^31^ FBXO38, specifically, is a coactivator of the Kruppel-like factor 7 (KLF7) zinc-finger transcription factor.^32^^,^^33^ While little is known about the function of FBXO38 and its potential role in COPD, the lack of the F box protein, which is responsible for protein-protein interactions, in disease-associated isoforms indicates that FBXO38 interactions are critical for protection against COPD.

We identified an additional colocalization between the COPD GWAS and a previously unidentified candidate gene, *BTC*. We specifically found that rs62316278, the lead GWAS SNP in the *BTC* region, is also associated with splicing of exon 4, with the risk allele (C) increasing exon 4 inclusion. The primary form of *BTC*, GenBank: NM_001729, includes exon 4, and rs62316278-C is associated with an increased proportion of GenBank: NM_001729 relative to GenBank: NM_001316963, a protein-coding isoform that lacks exon 4. This suggests that reduced inclusion of exon 4 is protective for COPD. BTC is a member of the epidermal growth factor (EGF) family of peptide ligands and a ligand for EGF receptor (EGFR). Human *BTC* encodes a 178 amino acid product corresponding to the BTC precursor protein (pro-BTC) and contains several domains including a signal peptide, an EGF motif, and transmembrane domains.^34^ The mature sequence of BTC is cleaved from the extracellular domain of BTC to produce an 80 amino acid protein. Based on the structure of other members of the EGF family, exon 4 is predicted to make up the third loop of the EGF-like motif and the transmembrane domain.^34^ The EGF domain is critical to binding with EGF ligands, and therefore, the isoform lacking exon 4 could be predicted to have altered function. Several previous studies have linked *BTC* expression to COPD, and *BTC* has been found to be higher in ex-smokers with COPD than those without COPD, has been associated with emphysema in alpha-1 antitrypsin deficiency,^35^ and has been found to be elevated in acute exacerbations in COPD.^36^ More targeted work is needed to investigate the function of *BTC* isoforms in COPD.

The major strength of this study is the large sample size, which allowed us to comprehensively characterize alternative splicing in whole blood and lung tissue. Our sample size significantly exceeds that of other commonly used resources, such as the GTEx, which includes 515 lung samples and 670 blood samples. While we sought colocalization with Global Initiative for Chronic Obstructive Disease (GOLD)-defined COPD, we expect that other respiratory- or smoking-related genetic associations would benefit from the use of this resource. One potential weakness is that our RNA-seq was performed using whole-lung tissue and whole-blood samples, which contain a variety of cell types. Therefore, some of the changes in transcriptional splicing detected may actually be reflecting differences in inter-individual cell proportions. Another limitation is the use of Moloc, which attempts colocalization with the most significant signal in the region and may not be optimal in the setting of multiple QTL or GWAS signals in the region. In addition, many GWAS loci colocalized with multiple QTL types and with multiple genes. While we characterized the colocalization with the highest PPA for each locus, it is possible that some loci can be explained by multiple genes and mechanisms. A full list of all colocalizations with all QTL types is shown in Table S2. Comparisons of the number of QTLs or the number of colocalizations between lung tissue and blood tissue may be influenced by the different library preparation methodology used for COPDGene blood compared to LTRC lung RNA-seq data. In COPDGene, total RNA was sequenced, which allows for the inclusion of transient and incompletely processed RNA, while poly(A) selection was used in LTRC. Additional work characterizing splicing using single-cell short- or long-read RNA-seq is required.

In conclusion, we discovered that multiple COPD GWAS associations colocalize with sQTLs and identified or replicated several candidate genes as COPD targets for follow-up.

## Acknowledgments

This work was funded by NIH grants K01HL157613, R01HL157879, P01HL114501, X01HL139404, R01HL124233, R01HL126596, R01HL153248, R01HL149861, R01 HL111527HL135142, and 10.13039/100000057NIGMS R35 GM140844. Molecular data for the Trans-Omics in Precision Medicine (TOPMed) program were supported by the 10.13039/100000050National Heart, Lung, and Blood Institute (NHLBI). Whole-genome sequencing and RNA-seq for "NHLBI TOPMed: The Lung Tissue Research Consortium (phs001662)” was performed at the Northwest Genome Center (NWGC; HHSN268201600032I, RNA-seq), and broad genomics (HHSN268201600034I, WGS) core support, including centralized genomic read mapping and genotype calling, along with variant quality metrics and filtering, were provided by the TOPMed Informatics Research Center (3R01HL-117626-02S1; contract HHSN268201800002I). Core support including phenotype harmonization, data management, sample-identity QC, and general program coordination were provided by the TOPMed Data Coordinating Center (R01HL-120393 and U01HL-120393; contract HHSN268201800001I). We gratefully acknowledge the studies and participants who provided biological samples and data for TOPMed. The COPDGene study (NCT00608764) is supported by grants from the 10.13039/100000050NHLBI (U01HL089897 and U01HL089856), by 10.13039/100000002NIH contract 75N92023D00011, and by the 10.13039/100008184COPD Foundation through contributions made to an industry advisory committee that has included AstraZeneca, Bayer Pharmaceuticals, Boehringer-Ingelheim, Genentech, GlaxoSmithKline, Novartis, Pfizer, and Sunovion. This study utilized biological specimens and data provided by the Lung Tissue Research Consortium (LTRC) supported by the 10.13039/100000050NHLBI.

## Author contributions

Conceptualization, A.S., P.J.C., and C.P.H.; data curation, M.H.C., P.J.C., C.P.H., C.V., and E.K.S.; formal analysis, A.S., W.K., and R.P.C.; writing – original draft preparation, A.S. and C.P.H.; writing – review & editing, A.S., W.K., R.P.C., C.V., E.K.S., M.H.C., P.J.C., and C.P.H.

## Declaration of interests

P.J.C. received grant support from Bayer and Sanofi and consulting fees from Verona Pharmaceuticals. C.P.H. has received research grants from Bayer, Boehringer-Ingelheim, and Vertex and consulting fees from Chiesi, Sanofi, and Takeda. M.H.C. has received grant funding from Bayer. In the past 3 years, E.K.S. received grant support from Bayer and Northpond Laboratories.

## Web resources

GenBank, https://www.ncbi.nlm.nih.gov/genbank/

## Supplemental information

Document S1. Figures S1–S3, Tables S1 and S2, and supplemental methods

Document S2. Article plus supplemental information
