# Supplementary material for: Overlap between COPD genetic association results and transcriptional quantitative trait loci
Source: HGG Adv. 2025 Aug 11;7(1):100493. doi: 10.1016/j.xhgg.2025.100493 (PMC12481885; doi:10.1016/j.xhgg.2025.100493)
Supplement: Document S1. Figures S1–S3, Tables S1 and S2, and supplemental methods [file mmc1.pdf]

HGGA, Volume *7*

Supplemental information

Overlap between COPD genetic association results and transcriptional quantitative trait loci

Aabida Saferali, Wonji Kim, Robert P. Chase, NHLBI TransOmics in Precision Medicine (TOPMed), Christopher Vollmers, Edwin K. Silverman, Michael H. Cho, Peter J. Castaldi, and Craig P. Hersh

Supplementary Results:

Identification of an additional colocalization between FBXO38 splicing and COPD GWAS

We found that rs10037493-T (5:148475407:C:T), which is associated with increased COPD risk is also associated with decreased inclusion of the identified exon (Supplementary Figure 1 a-b). This variant has a 71% probability of being causal for the sQTL association according to SuSie finemapping [23]. While long read sequencing did not identify a confirmed isoform containing this exon (ie in the isoforms passing filtering thresholds), in the raw RNAseq reads we identified at least 15 reads supporting the presence of this exon (Supplementary Figure 1c and Supplementary Figure 2). These reads correspond to two predicted protein-coding isoforms, XM_017009902 and XM_011537684, which would code for 713 and 788 amino acid proteins which differ from the usual 1188 amino acid protein

Supplementary Figures

Supplementary Figure 1

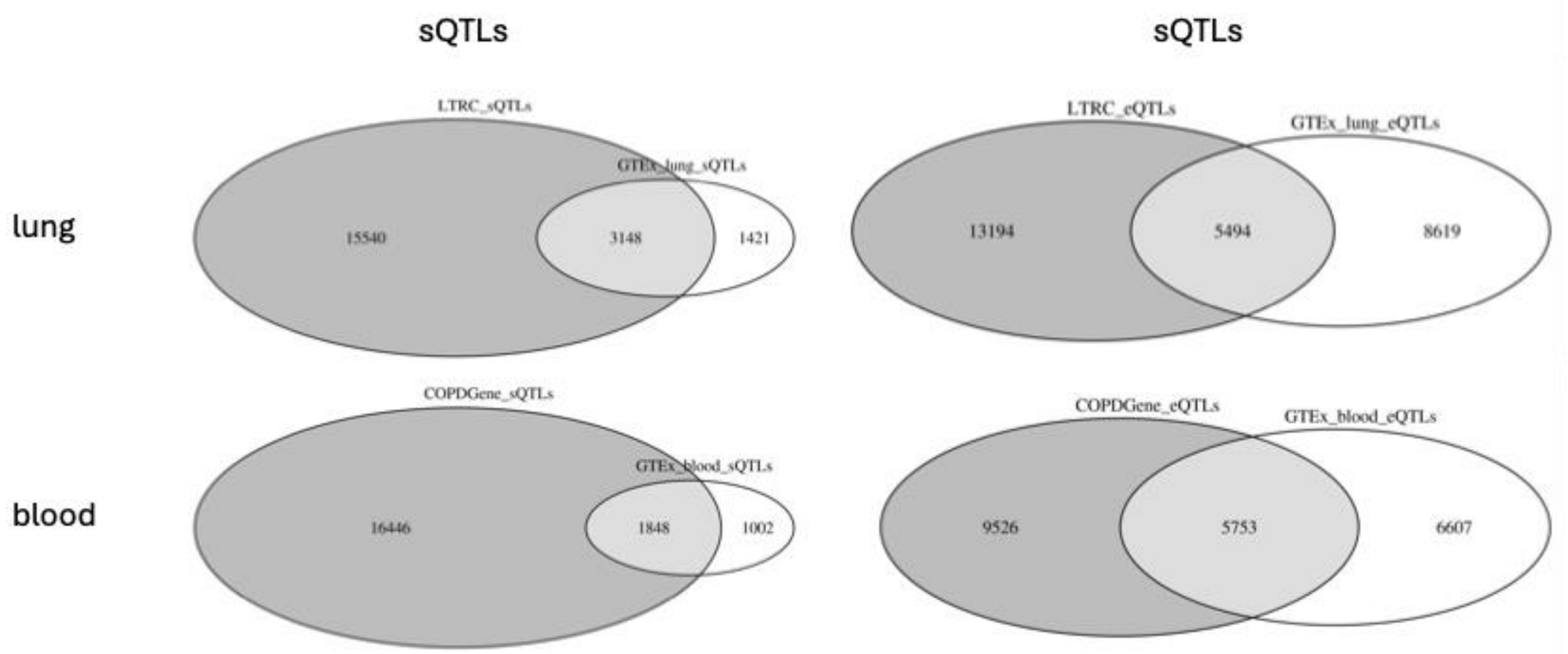

Supplementary Figure 1: Overlap between LTRC and COPDGene eQTLs and sQTLs with GTEx eQTLs and sQTL from lung tissue and blood. sQTL results are shown for splice clusters (instead of individual introns/junctions).

Supplementary Figure 2

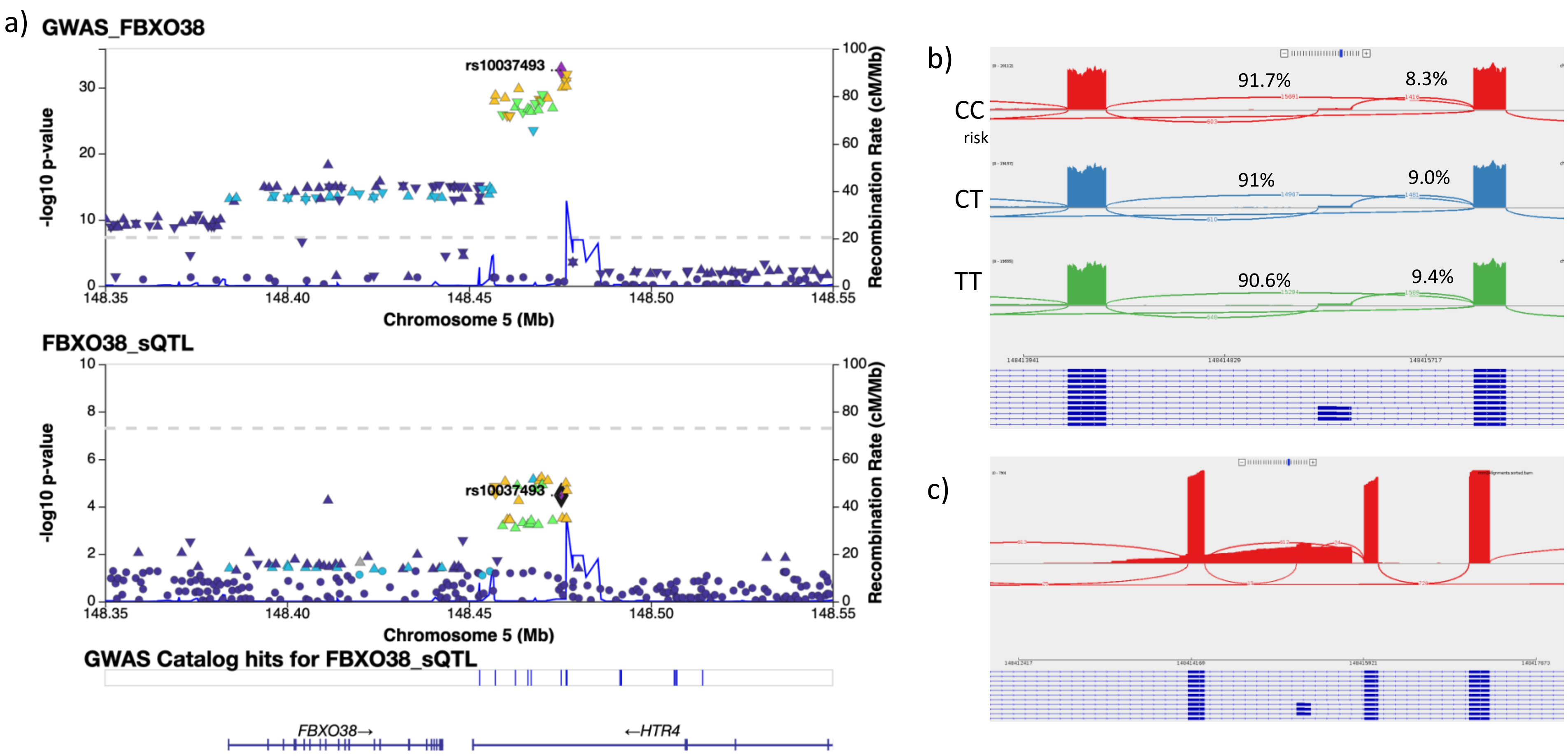

Supplementary Figure 2: An additional colocalization between *FBXO38* sQTLs and COPD. a) Locus association plot for COPD GWAS and *FBXO38* sQTL. The lead colocalized SNP, rs10037493, is highlighted in purple and used as the LD reference. b) IGV sashimi plot showing the region spanning chr5:148413941-148416605 for 273 subjects from each genotype of rs10037493. c) Long read sequencing data showing raw FBXO38 reads of isoforms containing the exon of interest.

Supplementary Figure 3

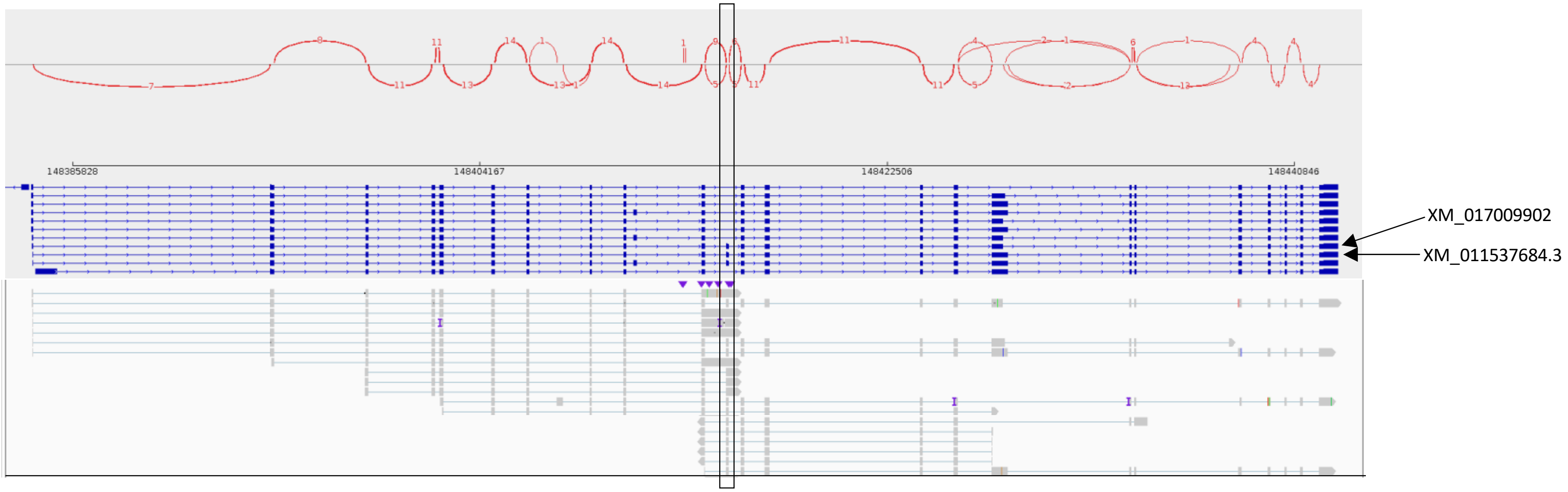

Supplementary Figure3: IGV plot of raw long read sequencing data from FBXO38 showing reads containing the exon

Supplementary Table 1: 82 COPD GWAS loci identified in previous studies

lead GWAS variant	lead variant rsID	window variant	Colocalization window start	colocalization window end	Lead variant effect allele	Lead variant beta	Lead variant p
1:16979534:C:A	rs9435731	1:16979534:C:A	15979534	17979534	A	0.0613	6.55E-10
1:39594353:G:A	rs76841360	1:39594353:G:A	38594353	40594353	A	0.0728	4.95E-10
1:45480964:G:T	rs4660861	1:45480964:G:T	44480964	46480964	T	-0.0549	4.42E-08
1:60447471:C:T	rs72673419	1:60447471:C:T	59447471	61447471	T	0.1299	4.00E-09
1:111195486:C:T	rs629619	1:111195486:C:T	110195486	112195486	T	0.078	2.94E-10
1:218515813:T:C	rs3009947	1:218515813:T:C	217515813	219515813	T	-0.0567	4.02E-09
1:219751552:T:A	rs11118406	1:219751552:T:A	218751552	220751552	A	-0.0725	4.11E-11
1:239737706:G:C	rs11579382	1:239737706:G:C	238737706	240737706	C	0.0582	6.50E-09
2:9150228:C:T	rs955277	2:9150228:C:T	8150228	10150228	T	0.0652	1.90E-10
2:15766055:C:T	rs10929386	2:15766055:C:T	14766055	16766055	T	-0.0573	9.11E-09
2:42206107:C:T	rs12466981	2:42206107:C:T	41206107	43206107	T	-0.061	4.85E-08
2:156156523:C:T	rs72902175	2:156156523:C:T	155156523	157156523	T	0.0824	1.63E-08
2:217818431:A:G	rs2571445	2:217818431:A:G	216818431	218818431	A	0.0701	3.43E-12
2:228705203:C:G	rs16825267	2:228705203:C:G	227705203	229705203	C	0.1762	1.75E-20
2:238951008:C:T	rs62191105	2:238951008:C:T	237951008	239951008	T	-0.089	2.58E-12
3:11599127:G:A	rs2442776	3:11599127:G:A	10599127	12599127	A	-0.0879	1.99E-10
3:25479091:C:A	rs1529672	3:25479091:C:A	24479091	26479091	A	-0.0867	2.54E-11
3:29430921:G:C	rs13073544	3:29430921:G:C	28430921	30430921	C	0.062	2.04E-08
3:55124197:A:G	rs17759204	3:55124197:A:G	54124197	56124197	A	-0.0713	8.81E-11
3:57760788:C:T	rs62259026	3:57760788:C:T	56760788	58760788	T	-0.0647	2.44E-08
3:123358195:T:A	rs4093840	3:123358195:T:A	122358195	124358195	A	0.0621	3.91E-10
3:128242335:T:A	rs2955083	3:128242335:T:A	127242335	129242335	A	0.1224	3.53E-15
3:141428572:T:C	rs7650602	3:141428572:T:C	140428572	142428572	T	-0.0546	4.86E-08
3:169028357:G:A	rs7642001	3:169028357:G:A	168028357	170028357	A	0.078	1.08E-14
4:74748153:G:A	rs4585380	4:74748153:G:A	73748153	75748153	A	-0.0665	3.40E-09
4:88962667:G:A	rs7671261	4:88962667:G:A	87962667	89962667	A	0.0855	1.43E-18
4:105897896:G:A	rs34712979	4:105897896:G:A	104897896	106897896	A	0.1691	3.04E-46
4:144567946:A:G	rs13140176	4:144567946:A:G	143567946	145567946	A	0.1662	4.09E-59
5:52899203:G:A	rs1551943	5:52899203:G:A	51899203	53899203	A	0.0725	5.83E-10
5:72848178:C:T	rs34651	5:72848178:C:T	71848178	73848178	T	-0.1012	3.02E-08
5:95700996:C:T	rs153916	5:95700996:C:T	94700996	96700996	T	0.0602	6.32E-10
5:133103318:T:A	rs62375246	5:133103318:T:A	132103318	134103318	A	0.0626	2.22E-08
5:148475407:C:T	rs10037493	5:148475407:C:T	147475407	149475407	T	-0.1189	2.58E-33
5:151215512:A:G	rs979453	5:151215512:A:G	150215512	152215512	A	-0.0598	1.40E-08
5:157510035:A:G	rs10866659	5:157510035:A:G	156510035	158510035	A	-0.0849	1.17E-16
5:171474582:A:T	rs12519165	5:171474582:A:T	170474582	172474582	A	0.0653	1.06E-09

6:7211585:G:A	rs1334576	6:7211585:G:A	6211585	8211585	A	0.0555	1.19E-08
6:19842430:C:T	rs9350191	6:19842430:C:T	18842430	20842430	T	0.1113	5.11E-14
6:22004680:C:T	rs13198656	6:22004680:C:T<br>6:25417423:G:T<br>6:26509382:C:T<br>6:27669976:A:G<br>6:28712247:A:G	21004680<br>24417423<br>25509382<br>26669976<br>27712247	23004680<br>26417423<br>27509382<br>28669976<br>29712247	T	0.0608	1.23E-09
6:30745803:T:C	rs2284174	6:30745803:T:C	29745803	31745803	T	-0.1174	2.10E-21
6:32183666:C:T	rs2070600	6:32183666:C:T<br>6:32628407:T:C<br>6:33710967:C:A	31183666<br>31628407<br>32710967	33183666<br>33628407<br>34710967	T	-0.1865	1.09E-17
6:108945052:T:C	rs2806356	6:108945052:T:C	107945052	109945052	T	-0.0977	2.89E-15
6:116935855:T:C	rs674621	6:116935855:T:C	115935855	117935855	T	-0.0603	7.61E-09
6:139959261:T:C	rs646695	6:139959261:T:C	138959261	140959261	T	-0.0747	4.62E-11
6:142347764:T:C	rs9399401	6:142347764:T:C	141347764	143347764	T	0.147	1.64E-40
7:2712518:G:A	rs798565	7:2712518:G:A	1712518	3712518	A	-0.0654	3.93E-09
7:20378511:C:T	rs2040732	7:20378511:C:T	19378511	21378511	T	-0.0581	6.89E-09
7:100032719:C:T	rs2897075	7:100032719:C:T	99032719	101032719	T	-0.0636	7.26E-10
8:8840148:C:G	rs9329170	8:8840148:C:G	7840148	9840148	C	0.0924	3.56E-10
9:4143749:A:T	rs10114763	9:4143749:A:T	3143749	5143749	A	-0.0711	8.66E-13
9:23588686:T:C	rs156394	9:23588686:T:C	22588686	24588686	T	0.0702	3.79E-12
9:82511248:T:C	rs7866939	9:82511248:T:C	81511248	83511248	T	-0.0593	1.75E-08
9:98899368:G:A	rs10760580	9:98899368:G:A	97899368	99899368	A	-0.0717	1.18E-10
9:116639371:G:A	rs803923	9:116639371:G:A	115639371	117639371	A	0.0546	2.72E-08
10:12235993:C:T	rs7068966	10:12235993:C:T	11235993	13235993	T	-0.0959	6.22E-23
10:76559121:A:C	rs2579762	10:76559121:A:C	75559121	77559121	A	-0.0625	2.58E-10
10:79946568:A:G	rs721917	10:79946568:A:G	78946568	80946568	A	-0.0544	2.21E-08
10:103897116:G:A	rs1570221	10:103897116:G:A	102897116	104897116	A	0.0575	2.22E-08
11:13149689:C:T	rs4757118	11:13149689:C:T	12149689	14149689	T	0.059	3.79E-09
11:86733719:A:G	rs117261012	11:86733719:A:G	85733719	87733719	A	-0.0837	6.91E-10
12:28167603:T:A	rs11049386	12:28167603:T:A	27167603	29167603	A	-0.0618	2.70E-08
12:95843792:T:C	rs7307510	12:95843792:T:C	94843792	96843792	T	-0.0736	2.64E-09
12:115510096:A:G	rs7958945	12:115510096:A:G	114510096	116510096	A	-0.0627	1.03E-09
13:44268367:G:A	rs9525927	13:44268367:G:A	43268367	45268367	A	-0.0735	2.78E-09
14:92639608:G:C	rs72699855	14:92639608:G:C<br>14:94349487:T:C	91639608<br>93349487	93639608<br>95349487	C	-0.0749	4.82E-09
15:49692513:G:C	rs72731149	15:49692513:G:C	48692513	50692513	C	-0.1114	8.27E-09
15:71320175:T:G	rs1441358	15:71320175:T:G	70320175	72320175	T	-0.1208	7.39E-33
15:78606590:C:G	rs55676755	15:78606590:C:G	77606590	79606590	C	-0.1088	2.75E-26
15:83724155:G:A	rs10152300	15:83724155:G:A	82724155	84724155	A	-0.081	4.20E-12
16:10615156:T:C	rs56134392	16:10615156:T:C	9615156	11615156	T	-0.057	4.51E-08
16:57988721:G:A	rs8044657	16:57988721:G:A	56988721	58988721	A	-0.1032	1.12E-08
16:75306333:A:T	rs4888379	16:75306333:A:T	74306333	76306333	A	-0.0926	5.86E-21

17:30086111:T:C	rs8080772	17:30086111:T:C	29086111	31086111	T	0.0593	1.37E-08
17:38678826:C:T	rs34727469	17:38678826:C:T	37678826	39678826	T	0.0827	1.07E-08
17:40062520:G:A	rs62065216	17:40062520:G:A	39062520	41062520	A	0.0586	8.07E-09
17:45846834:C:G	rs12373142	17:45846834:C:G	44846834	46846834	C	-0.073	9.94E-10
17:71220546:T:C	rs11655567	17:71220546:T:C	70220546	72220546	T	-0.0588	1.90E-09
18:8808466:T:C	rs647097	18:8808466:T:C	7808466	9808466	T	-0.0742	1.03E-11
19:45790878:G:A	rs72626215	19:45790878:G:A	44790878	46790878	A	-0.0685	1.67E-09
21:34289445:A:C	rs2096468	21:34289445:A:C	33289445	35289445	A	0.0541	4.00E-08
22:18006117:G:C	rs9617650	22:18006117:G:C	17006117	19006117	C	-0.079	4.44E-10
22:32939401:C:G	rs73158393	22:32939401:C:G	31939401	33939401	C	0.0666	7.67E-09

Supplementary Table 2: Additional colocalization signals

Lead GWAS variant	Best colocalized SNP	Locus	Best colocalized Gene	Best Colocalized Type	Other colocalized genes	GWAS identified target gene
4:144567946:A:G	4:144567946:A:G	4q31.21	HHIP	LTRC_sQTL	LTRC_eQTL_HHIP_0.9	HHIP
4:105897896:G:A	4:105897896:G:A	4q24	NPNT	LTRC_sQTL		PPA2, NPNT
5:148475407:C:T	5:148475407:C:T	5q32	FBXO38	LTRC_sQTL	COPDGene_sQTL_ABLIM3_0.89	FBXO38
15:71320175:T:G	15:71329185:G:A	15q23	THSD4	LTRC_eQTL	LTRC_eQTL_THSD4_0.99	THSD4, LARP6
16:75306333:A:T	16:75439564:G:A	16q23.1	TMEM170A	COPDGene_sQTL	COPDGene_sQTL_CFDP1_0.8	CFDP1, TERF2IP, CTRB2
6:30745803:T:C	6:30814428:G:C	6p21.33	IER3	COPDGene_sQTL	LTRC_sQTL_FLOT1_0.97<br>LTRC_sQTL_PPT2-EGFL8_0.85<br>LTRC_sQTL_NRM_0.8	IER3, FLOT1, TUBB, DDR1, VARS2 ,SFTA2, CDSN
4:88962667:G:A	4:88948181:T:C	4q22.1	PKD2	COPDGene_sQTL		FAM13A, NAP1L5,PPM1K
6:32183666:C:T	6:32660630:T:C	6p21.32	.	COPDGene_sQTL	LTRC_eQTL_AGER_0.99<br>LTRC_sQTL_PSORS1C1_0.95<br>COPDGene_sQTL_CCHCR1_0.95<br>COPDGene_eQTL_VARS2_0.94<br>COPDGene_sQTL_VARS2_0.94<br>LTRC_sQTL_NOTCH4_0.93<br>LTRC_sQTL_LST1_0.93<br>LTRC_sQTL_AIF1_0.92<br>COPDGene_sQTL_AGER_0.9<br>LTRC_sQTL_CCHCR1_0.88<br>LTRC_sQTL_SFTA2_0.86<br>LTRC_sQTL_VARS2_0.8	AGER, NOTCH4, AGPAT1, CYP21A2, MSH5, LY6G6E HLA-DQB1
5:157510035:A:G	5:157505976:A:T	5q33.3	ADAM19	LTRC_eQTL	COPDGene_sQTL_ADAM19_0.8 9	ADAM19, CYFIP2, CLINT1, SOX30, C5orf52
3:128242335:T:A	3:128242335:T:A	3q21.3	EEFSEC	COPDGene_sQTL	COPDGene_sQTL_RUVBL1_0.91	RUVBL1, GATA2
2:238951008:C:T	2:238965524:G:A	2q37.3	TWIST2	LTRC_eQTL		UBE2F
3:25479091:C:A	3:25496178:G:A	3p24.2	RARB	LTRC_eQTL		RARB

2:9150228:C:T	2:9145396:G:A	2p25.1	LINC00299	COPDGene_sQTL		ASAP2
1:16979534:C:A	1:16979534:C:A	1p36.13	CROCC	COPDGene_sQTL	LTRC_sQTL_MST1P2_0.98<br>LTRC_eQTL_ATP13A2_0.98<br>LTRC_sQTL_CROCC_0.98<br>LTRC_sQTL_MFAP2_0.98<br>LTRC_sQTL_CROCCP2_0.97<br>LTRC_eQTL_MFAP2_0.96<br>LTRC_APAQTL_MFAP2_0.82	MFAP2<br>PADI2
7:100032719:C:T	7:100032719:C:T	7q22.1	ZSCAN21	COPDGene_sQTL	COPDGene_eQTL_MCM7_0.98<br>COPDGene_APAQTL_ZKSCAN1_0.97<br>COPDGene_APAQTL_STAG3L5P_0.96<br>COPDGene_sQTL_CNPY4_0.95<br>LTRC_sQTL_COPS6_0.93<br>LTRC_sQTL_ZSCAN21_0.8	ZKSCAN1, ZNF3, AZGP1, AP4M1, CNPY4, TRIM4, PVRIG, NYAP1
19:45790878:G:A	19:45790878:G:A	19q13.32	DMWD	LTRC_APAQTL	LTRC_APAQTL_SYMPK_0.97<br>LTRC_sQTL_QPCTL_0.82	DMPK, SNRPD2
1:39594353:G:A	1:39582337:G:A	1p34.3	PPIEL	COPDGene_sQTL	COPDGene_sQTL_MACF1_0.88<br>COPDGene_sQTL_PABPC4_0.85	PABPC4, OXCT2, MYCL
12:95843792:T:C	12:95843792:T:C	12q23.1	NTN4	LTRC_sQTL	COPDGene_sQTL_CFAP54_0.88	AMDHD1
4:74748153:G:A	4:74748514:C:T	4q13.3	BTC	LTRC_sQTL		EPGN
1:111195486:C:T	1:111195294:T:C	1p13.3	DENND2D	COPDGene_sQTL	LTRC_eQTL_KCNA2_0.84	DRAM2, OVGP1, INKA2
14:92639608:G:C	14:92649065:G:C	14q32.12	RIN3	LTRC_APAQTL		CPSF2, RIN3, ITPK1
1:239737706:G:C	1:239689643:G:C	1q43	CHRM3-AS2	COPDGene APAQTL	COPDGene_sQTL_CHRM3-AS2_0.95	
11:13149689:C:T	11:13145018:T:C	11p15.2	RASSF10	LTRC_eQTL		PARVA

5:151215512:A:G	5:151215512:A:G	5q33.1	GM2A	LTRC_APAQTL	COPDGene_APAQTL_GM2A_0.95	
7:2712518:G:A	7:2830820:T:G	7p22.3	GNA12	LTRC_sQTL		GNA12
17:38678826:C:T	17:38730575:G:A	17q12	CISD3	LTRC_eQTL	LTRC_eQTL_PCGF2_0.94<br>COPDGene_eQTL_MLLT6_0.94<br>LTRC_eQTL_EPOP_0.92<br>COPDGene_sQTL_MLLT6_0.9<br>COPDGene_eQTL_AC006449.6_0.88	CACNB1
3:29430921:G:C	3:29430921:G:C	3p24.1	RBMS3	LTRC_eQTL		RBMS3
15:49692513:G:C	15:49578340:A:G	15q21.2	FAM227B	LTRC_sQTL	COPDGene_sQTL_FAM227B_0.92<br>COPDGene_sQTL_AP4E1_0.9	FAM227B, SLC27A2
16:57988721:G:A	16:58063696:G:C	16q21	MMP15	LTRC_sQTL		CNGB1
10:79946568:A:G	10:80458350:G:C	10q22.3	TSPAN14	COPDGene_sQTL	COPDGene_sQTL_FAM213A_0.95<br>LTRC_APAQTL_SFTPA2_0.89<br>LTRC_sQTL_DYDC2_0.89	TMEM254, DYDC2, FAM213A
1:45480964:G:T	1:45543641:G:C	1p34.1	TESK2	COPDGene APAQTL	COPDGene_eQTL_AKR1A1_0.95<br>COPDGene_sQTL_AKR1A1_0.95<br>COPDGene_APAQTL_MUTYH_0.93<br>LTRC_sQTL_AKR1A1_0.87	MUTYH, IPP, UROD
2:42206107:C:T	2:42206107:C:T	2p21	COX7A2L	COPDGene APAQTL		EML4
17:30086111:T:C	17:30129740:C:T	17q11.2	NSRP1	COPDGene_sQTL	LTRC_sQTL_NSRP1_0.82	SLC6A4, EFCAB5, GOSR1 CORO6

<u>Supplementary Methods</u>: Long Read Sequencing

cDNA synthesis

100 ng of RNA was mixed with dNTP and RNA Inhibitor, then denatured to remove secondary structure for 3 minutes at 72C. Prior to reverse transcription (RT), indexed oligo(dT) primers were added to the mix at 50C. First strand synthesis using Maxima H(-) reverse transcriptase (Thermo Fisher) and a template switching oligo was performed for 90 minutes at 50C. The cDNA was then amplified using PCR with SeqAmp polymerase, ISPCR primer, and Nextera A index primer (95C for 1 minute, 15 cycles of 98C for 15 seconds, 65C for 30 seconds, 68C for 6 minutes, 72C for 7 minutes, 4C hold). Each sample was indexed using a unique combination of olgo(dT) primer and Nextera A index primer. cDNA was cleaned up using ProNex Size Selective Purification System (Promega) at a 1:1.2 ratio.

Enrichment

After quantification by Qubit, equal amounts cDNA from every sample were pooled together. 3 ug of cDNA pool were target enriched using the xGEN Hybridization Capture (Integrated DNA Technologies). xGen Human Cot DNA and Blocking Oligos were added to the cDNA pool and cleaned using SPRI beads at a 1.8:1 ratio. The beads were eluted in a mix of xGen 2X Hybridization Buffer, xGen Hybridization Buffer Enhancer, and xGen Hyb Panel and incubated at room temperature for 5 min. The beads were removed using a magnet, and the solution incubated at 95C for 30 seconds, 65C for 4 hours, and a 65C hold. Streptavidin beads were added and incubated at 65C for 45 minutes. The mixture was washed using Wash Buffer 1 and Stringent Wash Buffer at 65C. Then, Wash Buffer 1, Wash Buffer 2, and Wash Buffer 3 were used to wash the mixture at room temperature. PCR was performed on the final solution with SeqAmp polymerase, ISPCR primer, and Nextera A Universal primer for 15 cycles (95C for 1 minute, 98C for 15 seconds, 65C for 30 seconds, 68C for 6 minutes, 72C for 7 minutes, 4C hold). The final enriched library was cleaned up using SPRI beads at 0.65:1 ratio.

R2C2 library preparation and sequencing

DNA splints were generated through primer extension as previously described using 2 complementary DNA oligos. Enriched cDNA libraries were circularized by Gibson assembly (NEBbuilder HiFi) with a DNA splint with ends complementary to the cDNA. ExoI, ExoIII, and Lambda Exonuclease (NEB) were added and incubated for 16 hours at 37C to remove non-circular molecules followed by heat inactivation for 20 minutes at 80C. The reaction was then cleaned using SPRI beads at a 0.8:1 ratio. The clean, circularized library was used as the template for rolling circle amplification (RCA) using Phi29 (NEB) with a random hexamer primer for 18 hours at 30C, and then heat inactivated for 10 minutes at 65C. The reaction was then debranched using T7 endonuclease (NEB) for 2 hours at 37C before being cleaned and concentrated using a Monarch PCR and DNA cleanup kit (NEB).The resulting R2C2 DNA was then run on a 1% agarose gel and DNA >10kb was excised and purified using a Monarch DNA Gel extraction kit (NEB).

Following the manufacturer's protocol, we then used the Genomic DNA by Ligation (SQK-LS114) kit from ONT to generate sequencing libraries from this size-selected R2C2 DNA. The final library was loaded onto a PromethION R10.4 flow cell and run at 400bp/s. Approximately once per day, flow cells were flushed and treated with DNAse I, then loaded with additional library to increase sequencing throughput.

Resulting raw reads were basecalled using the SUP model of guppy (v6) and consensus called and demultiplexed using C3POa (v2.3).

Oligos used:

cDNA synthesis

```
ISPCR primer        AAGCAGTGGTATCAACGCAGAGTAC
Oligo_dT_Index1     AAGCAGTGGTATCAACGCAGAGT [10nt index] ACTTTTTTTTTTTTTTTTTTTTTTTTTTTTTTVN
TSO1_Nextera        TCGTCGGCAGCGTCAGATGTGTATAAGAGArCAG  rUGA  ArU  rUC  TGGTrGrGrG
```

```
Nextera_Primer_A1       AATGATACGGCGACCACCGAGATCTACAC  [8nt index]  TCGTCGGCAGCGTCAGATG
```

Enrichment

```
NextA_F_Blocking        AATGATACGGCGACCACCGAGATCTACAC  IIIIIIII  TCGTCGGCAGCGTCAGATGTGTATAAGAGACAG/3ddC/
NextA_RC_Blocking       CTGTCTCTTATACACATCTGACGCTGCCGACGA  IIIIIIII  GTGTAGATCTCGGTGGTCGCCGTATCATT
RC_OligodT              AA AAA AAA AAA AAA AAA AAA AAA AAA AAA AGT III III III IAC TCT GCG TTG ATA CCA CTG CTT
IndexedOligodTBlocker   AAG CAG TGG TAT CAA CGC AGA GTI III III III ACT TTT TTT TTT TTT TTT TTT TTT TTT TTT TT/3ddC/
```

Splint Synthesis

```
Nextera_A_Splint_1_F    GATCTCGGTGGTCGCCGTATCATT TGAGGCTGATGAGTTCCATA NNNNNTATATNNNNN ATCACTACTTAGTTTTTGATAGCTTCAAGCCAGAGTTGTCTTTTTCTCTTTGCTGGCAGTAAAAG
ISPCR_Splint_1_R        ACTCTGCGTTGATACCACTGCTT AAAGGGATATTTTCGATCGC NNNNNATATANNNNN TTAGTGCATTTGATCCTTTTACTCCTCCTAAAGAACAACCTGACCCAGCAAAAGGTACACAATACTTTTACTGCCAGCAAAGAG
Nextera_A_Splint_2_F    GATCTCGGTGGTCGCCGTATCATT TGCCGGTTGGGTATCAATAA NNNNNTATATNNNNN ATTGCCTTTATTCTATCTACTTAGTTTTGGCGATGTAGTCTACCTATCCTGATGCTGAATAAAGGC
ISPCR_Splint_2_R        ACTCTGCGTTGATACCACTGCTT AATTAGGTTCTAGGATCACG NNNNNATATANNNNN CTGCCATCGAAAATTTTTCACCCGTAACAAGAACTTACAACTCTCTGACGCCTATATCATGAAGGCCTTTATTCAGCATCAGGA
Nextera_A_Splint_3_F    GATCTCGGTGGTCGCCGTATCATT GTCGTGATCAAACATTGGGT NNNNNTATATNNNNN TAAAAGTTTTCTGTGTCCATTACGTTTTTTGGAGACGGTCTCAACTATTCTTAATCTCGGCGAACT
ISPCR_Splint_3_R        ACTCTGCGTTGATACCACTGCTT AAGTGAGTCGTATTACAGCT NNNNNATATANNNNN ATATTCAGTTCGAAACCTTACCTCCTCTGAAAAGTGTCCCCGAAATCTCTAGGAAATTAACCCAAGTTCGCCGAGATTAAGAAT
Nextera_A_Splint_4_F    GATCTCGGTGGTCGCCGTATCATT TAGTGTAAGGTAGCATCCGT NNNNNTATATNNNNN TACGATCATATTATGTTCGTCTGTCTTTAACGGGCTATACTTCTTCTTTAGGAAAGGTCTGAACCT
ISPCR_Splint_4_R        ACTCTGCGTTGATACCACTGCTT CTACTATTTGAGTAAAGGCG NNNNNATATANNNNN ACCTGTGTATTAGACCCCACTACTACCCTTAAGATTCCATAACCTTAAGTAGAACCCAGATTGAAGGTTCAGACCTTTCCTAAA
```
