# Supplementary material for: Overlap between COPD genetic association results and transcriptional quantitative trait loci
Source: HGG Adv. 2025 Aug 11;7(1):100493. doi: 10.1016/j.xhgg.2025.100493 (PMC12481885; doi:10.1016/j.xhgg.2025.100493)
Supplement: Document S2. Article plus supplemental information [file mmc2.pdf]

HGG Advances

ARTICLE

# Overlap between COPD genetic association results and transcriptional quantitative trait loci

Aabida Saferali,[1,5,*] Wonji Kim,[1] Robert P. Chase,[1] NHLBI TransOmics in Precision Medicine (TOPMed), Christopher Vollmers,[2] Edwin K. Silverman,[1,3] Michael H. Cho,[1,3] Peter J. Castaldi,[1,4] and Craig P. Hersh[1,3]

## Summary

Genome-wide association studies (GWASs) have identified multiple genetic loci associated with chronic obstructive pulmonary disease (COPD). Here, we identify SNPs that are associated with alternative splicing (splicing quantitative trait loci [sQTLs]) and gene expression (expression QTLs [eQTLs]) to identify functions for COPD-associated genetic variants. RNA sequencing on whole blood from 3,743 subjects in the COPDGene Study and from lung tissue of 1,241 subjects from the Lung Tissue Research Consortium (LTRC) was analyzed. Associations between all SNPs within 1,000 kb of a gene (*cis*-) and splice and gene expression quantifications were tested using tensorQTL. We assessed colocalization with COPD-associated SNPs from a published GWAS. After adjustment for multiple statistical testing, we identified 28,110 splice sites corresponding to 3,889 unique genes that were significantly associated with genotype in COPDGene whole blood and 58,258 splice sites corresponding to 10,307 unique genes associated with genotype in LTRC lung tissue. To determine what proportion of COPD-associated SNPs were associated with transcriptional splicing, we performed colocalization analysis between COPD GWAS and sQTL data and found that 38 genomic windows, corresponding to 33 COPD GWAS loci, had evidence of colocalization between QTLs and COPD. The top five colocalizations between COPD and lung sQTLs include *Nephronectin* (*NPNT*), *F box protein 38* (*FBXO38*), *Hedgehog interacting protein* (*HHIP*), *Netrin 4* (*NTN4*), and *Betacellulin* (*BTC*). Overall, a total of 38 COPD GWAS loci contain evidence of sQTLs, suggesting that analysis of sQTLs in whole blood and lung tissue can provide insights into disease mechanisms.

## Introduction

Chronic obstructive pulmonary disease (COPD) is a complex disease characterized by irreversible airflow obstruction on lung function testing. The leading environmental risk factor for COPD is cigarette smoking; however, genetic factors have also been shown to play a role in disease susceptibility.[1–4] Genome-wide association studies (GWASs) have been used to identify genetic variants associated with COPD and lung function.[5,6–8] However, as for most complex trait GWAS associations, the causal mechanisms are currently unknown. While it has been found that expression quantitative trait loci (eQTL) are enriched among GWAS loci, a large proportion of disease heritability remains unexplained by eQTLs.[9] Previous work has shown that splicing QTLs (sQTLs), in which genetic variants affect alternative splicing, can account for at least as many GWAS loci as eQTLs.[10]

A recent GWAS for COPD, including 35,735 affected individuals and 222,076 control subjects from the UK Biobank and the International COPD Genetics Consortium, identified 82 loci associated with COPD with genome-wide significance.[5] Using S-PrediXcan,[11] the authors discovered that 49 GWAS loci had evidence for genetically regulated expression associated with COPD using data from the Lung-eQTL consortium.[5] As S-PrediXcan is also influenced by linkage disequilibrium (LD), most of the COPD GWAS loci are likely not explained using existing eQTLs.

Genomic loci identified as being eQTLs may also be sQTLs, as splicing is a common mechanism to alter total gene expression levels. In our previous work, we generated sQTLs in 376 subjects from the COPDGene study and found that these data could explain seven COPD GWAS associations, including the identification of *FBXO38* as a novel COPD susceptibility gene at 5q32.[10] Here, we expand upon our findings by developing a large database of eQTLs and sQTLs in RNA from lung tissue from 1,241 subjects and in RNA from blood from 3,743 subjects followed by colocalization analysis with COPD GWAS results.

## Material and methods

### Study population

This study included 3,713 non-Hispanic White and African American subjects from the COPDGene study and 1,241 subjects from the Lung Tissue Research Consortium (LTRC) (Table 1). COPDGene enrolled individuals between the ages of 45 and 80 years with a minimum of 10 pack-years of lifetime smoking history from 21 centers across the United States.[12] These subjects returned for a second study visit 5 years after the initial visit, at which time they completed additional questionnaires, pre- and

[1]Channing Division of Network Medicine, Brigham and Women's Hospital, Boston, MA, USA; [2]Department of Biomolecular Engineering, Cellular, Cellular, and Developmental Biology, University of California, Santa Cruz, Santa Cruz, CA, USA; [3]Division of Pulmonary and Critical Care Medicine, Brigham and Women's Hospital, Boston, MA, USA; [4]Division of General Medicine and Primary Care, Brigham and Women's Hospital, Boston, MA, USA
[5]Lead contact
*Correspondence: aabida.saferali@channing.harvard.edu
https://doi.org/10.1016/j.xhgg.2025.100493.
© 2025 The Authors. Published by Elsevier Inc. on behalf of American Society of Human Genetics.
This is an open access article under the CC BY license (http://creativecommons.org/licenses/by/4.0/).

**Table 1. Clinical characteristics of LTRC and COPDGene study individuals included in the analysis**

| | LTRC ($n = 1241$) | COPDGene ($n = 3734$) |
|---|---|---|
| Gender, male (%) | 52.4 | 51.1 |
| Age, mean (SD) | 63.3 (10.6) | 59.9 (8.7) |
| Race, *n* (%) | | |
| White | 1,118 (90.1) | 2,702 (72.4) |
| Asian | 4 (0.3) | – |
| Black | 81 (6.5) | 1,032 (27.6) |
| Hispanic | 25 (2.0) | – |
| Other | 13 (1.0) | – |
| Current smokers, *n* (%)[a] | 73 (5.9) | 1,766 (47.3) |
| Pack-years smoked, mean (SD) | 31.8 (±33.2) | 42.7 (±24.0) |

[a]Smoking data are missing for a subset of LTRC subjects.

post-bronchodilator spirometry, and computed tomography (CT) of the chest and provided blood for complete blood counts (CBCs) and RNA sequencing (RNA-seq). Samples were collected as part of the LTRC from individuals who were undergoing clinically indicated thoracic surgery procedures using a standardized protocol described in the original study design, which included pulmonary function testing, questionnaires, and chest CT. The COPDGene and LTRC studies have been approved by Mass General Brigham IRB (Protocol # 2007P000554 and 2018P000186).[13]

## RNA-seq, alignment, and count generation

The protocols for RNA-seq data generation and processing for COPDGene and LTRC have been previously described.[13,14] Briefly, for LTRC, mRNA sequencing (RNA-seq) was performed through the NHLBI TOPMed program at the University of Washington. Poly(A) selection and cDNA synthesis were performed using the TruSeq Stranded mRNA kit (Illumina, San Diego, CA), and sequencing was performed on the NovaSeq6000 instrument. Sequences were aligned to GRCh38 using STAR (v.2.6.1d) with the GENCODE release 29 reference. Gene-level expression quantification was performed using RSEM (v.1.3.1). For COPDGene, globin reduction, ribosomal RNA depletion, and cDNA library prep were performed on total RNA from whole blood using the TruSeq Stranded Total RNA with Ribo-Zero Globin kit (Illumina), and sequencing was performed on Illumina platforms. Sequences were aligned to GRCh38 using STAR 2-pass alignment (v.2.5.2b). Gene-level expression quantification was performed using Salmon (v.1.3.0) for pseudoalignment to the GENCODE release 37 transcriptome, followed by summarization of isoform-level counts to gene-level counts using tximport (v.1.8.5).[15,16] Quantification of splicing ratios was performed in COPDGene and LTRC using LeafCutter with default parameters.[17] Intron ratios were calculated by determining how many reads support a given exon-intron junction in relation to the number of reads in that region. LTRC RNA-seq data are available through TOPMed (https://topmed.nhlbi.nih.gov). COPDGene data are available on dbGaP with accession numbers phs000179 and phs000765.

## Whole-genome sequencing

All samples were sequenced through the TOPMed program. This analysis uses Freeze 8 data. LTRC genotyping data are available on dbGaP with accession number phs001662.v1.p1.

## eQTL and sQTL analysis

Gene expression counts were filtered to include only genes with at least 1 count per million in at least 20% of subjects, and the remaining counts were TMM normalized.[18] LeafCutter ratios were filtered to remove introns detected in less than 40% of individuals and introns with a standard deviation of less than 0.005 across subjects, and the remaining ratios were scale normalized (i.e., mean centered and divided by the standard error). TensorQTL[19] was used to test for association between genotypes of all SNPs within 1,000 kb of the gene boundary (*cis*-) and quantifications of splicing or gene expression using linear models, adjusting for gender, RNA-seq library preparation batch, principal components (PCs) of splicing (10 PCs) or gene expression data (170 PCs for COPDGene and 190 PCs for LTRC), and PCs of genetic ancestry (10 PCs). The calculation of PCs of genetic ancestry has been previously described.[20] A total of 8,792,206 variants (bi-allelic SNPs with a mean-allele frequency [MAF] > 0.01) were tested for association with 58,258 splice sites (corresponding to 10,615 unique genes) and expression of 16,264 genes. Results were annotated using ANNOVAR[21] with annotations derived from dbSNP build 150. Genotype Tissue Expression Project (GTEx) v.8 significant results[22] were obtained for all tissues from the GTEx Portal (https://www.gtexportal.org/home/datasets), and complete lung sQTL results were obtained from the Anvil GTEx Terra workspace.

## Colocalization analysis

Published GWAS data for COPD case-control status[5] were used for this analysis. Testing windows were generated by identifying all GWAS variants with $p < 1E^{-5}$ and calculating non-overlapping windows of 1 Mb on either side of each SNP. Only windows containing sQTLs or eQTLs with a false discovery rate (FDR) < 0.05 were tested. The 82 genome-wide significant loci and 89 testing windows are shown in Table S1. For each window, Bayesian colocalization tests were performed using the Moloc R package[23] to quantify the probability that the GWAS and sQTL or eQTL associations were due to a shared causal variant. Windows with a colocalization posterior probability (CPP) of greater than 0.8 were reported. Fine mapping was performed on QTL results from selected regions of interest using SuSieR v.12.35, with in-sample LD.[24]

**Table 2. Summary of eQTLs and sQTLs tested**

| | COPDGene | | LTRC | |
|---|---|---|---|---|
| | Genes | Splice sites | Genes | Splice sites |
| Before filtering | 60,232 | 237,155 | 58,962 | 306,475 |
| After filtering | 15,507 | 160,655 | 16,266 | 223,153 |
| Genes tested | 15,507 | 12,096 | 16,266 | 15,120 |
| SNPs tested | 11,869,333 | 11,869,333 | 8,792,206 | 8,792,206 |
| Significant QTLs[a] | 15,279 | 63,946 | 12,225 | 58,258 |

[a]The number of genes or splice sites significantly associated with at least one SNP with an FDR < 0.05.

### Long-read RNA-seq analysis in human lung samples from the LTRC

We conducted targeted Oxford Nanopore Technologies (ONT) long-read sequencing on RNA from 170 human lung samples from the LTRC on genes selected from colocalization analysis. The enrichment and library generation procedures are described in detail in the supplemental methods. The final library was loaded onto a PromethION R10.4 flow cell and run at 400 bp/s. Approximately once per day, flow cells were flushed and treated with DNase I and then loaded with an additional library to increase sequencing throughput. Resulting raw reads were base called using the SUP model of guppy (v.6) and consensus called and demultiplexed using C3POa (v.2.3). R2C2 reads were analyzed to identify and quantify isoforms using v.3.5 of Mandalorion (https://github.com/rvolden/Mandalorion-Episode-III). Mandalorian was run twice to identify high-abundance isoforms (>10% of total isoform expression; -O 0,40,0,40 -r 0.1 -i 1 -w 1 -n 2 -R 5) and high- and low-abundance isoforms (-O 0,40,0,40 -r 0.01 -i 1 -w 1 -n 2 -R 5).

## Results

### Quantification of gene expression and RNA splicing in COPDGene blood and LTRC lung

Using RNA-seq data from LTRC lung tissue ($n = 1{,}241$) and COPDGene whole blood ($n = 3{,}743$), we identified splice sites using LeafCutter,[17] which identifies and quantifies intron exclusion. After filtering out splice sites with low usage, we identified a total of 223,153 splice sites corresponding to 15,120 genes in LTRC and 160,655 splice sites corresponding to 12,096 genes in COPDGene (Table 2). In both LTRC and COPDGene, the majority (50.1% and 51.0%, respectively) of identified splice sites were annotated in GENCODE, followed by cryptic 3′, cryptic 5′, cryptic unanchored splice sites (meaning both splice donor and acceptor were unannotated), and novel annotated pairs (Table 3). The gene expression of 16,264 genes in LTRC and 15,507 genes in COPDGene met expression thresholds (Table 2).

### Identification of eQTL and sQTLs in human lung tissue and whole blood

We next tested for associations between genotype and gene expression or splicing to identify eQTLs and sQTLs in lung and blood. In LTRC lung tissue, we identified 58,258 splice sites (corresponding to 10,615 genes) associated with at least one SNP with $q < 0.05$; in COPDGene blood, 63,946 splice sites (corresponding to 9,433 genes) were associated with at least one SNP (Table 2). In addition, we identified 12,225 genes associated with at least one SNP (eQTLs) in LTRC and 15,279 eQTL genes in COPDGene. We next investigated what proportion of the QTLs found in our data were previously identified in GTEx lung ($n = 515$) and whole blood ($n = 670$). We found that out of 18,688 splice clusters that were significantly associated with at least one SNP in LTRC lung, 3,148 had sQTLs in GTEx lung, and of 18,294 COPDGene sQTL clusters, 1,848 were significant in GTEx blood. For eQTLs, 5,494 out of 12,225 LTRC eGenes were also eGenes in GTEx lung, and 5,753 out of 15,279 eGenes in COPDGene were also eGenes in GTEx blood (Figure S1).

We found that 14,946 sQTL splice-site-SNP pairs (13%) were found in both COPDGene blood and LTRC lung, while 6,349 eQTL gene-SNP pairs were shared between both tissues (25%) (Figure 1). In addition, we found that 5,787 sQTL gene-SNP pairs overlapped with eQTL gene-SNP pairs in LTRC (47%), while 7,455 sQTL gene-SNP pairs overlapped with eQTL gene-SNP pairs in COPDGene (49%).

### Functional annotation of sQTLs and eQTLs

Next, we categorized eQTLs and sQTLs-SNPs on the basis of their location relative to the gene with which they were associated (Table 4). The genomic distribution of eQTLs and sQTLs was similar, with the largest proportion of variants located upstream of the gene region in both COPDGene (32.1% and 30.1%, respectively) and LTRC (31.8% and 29.4%, respectively). The next most frequent SNPs were intronic, downstream of a gene, intergenic, and 3′ UTR variants. Only a small percentage of lead sQTL-SNPs directly modified a splice site. There was no difference in variant position in either eQTL vs. sQTL or COPDGene blood vs. LTRC lung.

### Colocalization of QTLs with COPD case-control GWAS data

We next sought to identify eQTLs and sQTLs that contribute to COPD risk by performing genetic

**Table 3. Annotations of LeafCutter splice sites identified in COPDGene and LTRC**

| | Frequency in LTRC | Percentage in LTRC | Frequency in COPDGene | Percentage in COPDGene |
|---|---|---|---|---|
| Annotated | 113,562 | 50.9 | 81,111 | 50.5 |
| Cryptic 5′ | 30,166 | 13.5 | 21,232 | 13.2 |
| Cryptic 3′ | 32,258 | 14.5 | 24,047 | 15.0 |
| Cryptic unanchored | 8,504 | 3.8 | 5,788 | 3.6 |
| Novel annotated pair | 17,991 | 8.1 | 13,293 | 8.3 |
| Unknown strand | 20,651 | 9.2 | 15,166 | 9.4 |

Annotated: both 5′ and 3′ splice sites have been previously annotated. Cryptic 5′: the 5′ splice site is not annotated, but the 3′ splice site is annotated. Cryptic 3′: the 3′ splice site is not annotated, but the 5′ splice site is annotated. Cryptic unanchored: neither splice site has been previously annotated. Novel annotated pair: both 5′ and 3′ splice sites have been individually annotated, but the combination has not been annotated as a junction. Unknown strand: it is not possible to determine the directionality of the splice sites based on the RNA-seq read.

colocalization between the QTL data and COPD case-control GWAS data. The 82 previously identified COPD GWAS loci[5] correspond to 89 windows of 2 Mb in width (Table S1). We also included colocalization results for 3′ UTR alternative polyadenylation QTLs (apaQTLs) from our previous study.[25] We identified 38 windows (corresponding to 32 of the 82 GWAS loci) with a colocalization posterior probability of association (PPA) > 0.8 with either eQTL, sQTL, or apaQTL data and GWAS $p < 5.0E-8$ (Table 5). We found that for 19 out of the 82 COPD loci, the strongest colocalization (largest PPA) with GWAS data was in LTRC, and for 19 loci, the strongest colocalization was in COPDGene. In LTRC, the largest number of GWAS loci colocalized with sQTLs (9 loci), followed by eQTLs (7 loci) and then apaQTLs (3 loci). In COPDGene, the majority of colocalizations were with sQTLs (15 loci), with only one 1 locus colocalizing most strongly with eQTLs and 3 with apaQTLs. We compared the colocalization findings with the target genes identified from the original GWAS analysis to determine whether the QTLs identified additional targets from previous analyses. We found that for 7 loci, all genes identified in the current QTL analysis were previously identified, and for 26 loci, one or more new targets were found (Table S2). For further characterization, we focused on sQTL colocalizations in LTRC lung, with the top five colocalizations (by highest PPA) being *Nephronectin* (*NPNT*), *F box protein 38* (*FBXO38*), *Hedgehog interacting protein* (*HHIP*), *Netrin 4* (*NTN4*), and *Betacellulin* (*BTC*). We have previously published on NPNT colocalizations in the lung,[26] and for HHIP, significant evidence suggests that the mechanism underlying the association is an eQTL effect.[27] *NTN4* appears to be a promoter-usage eQTL instead of an sQTL. Therefore, we highlight the results for *FBXO38* and *BTC* below. All colocalization results are available online at https://copd-moloc.bwh.harvard.edu/.

## Characterization of sQTL for FBXO38

We first sought to replicate the association we previously characterized in 365 subjects from COPDGene between the rs7730971 (GRCh38 chr5:148411297C>G) variant and *FBXO38* splicing in whole blood, which colocalized with COPD GWAS findings.[10] Here, we identified two splicing clusters (i.e., a group of splice junctions with shared start or stop positions[17]) in lung tissue in *FBXO38*, which were associated with COPD-related variants. First, we confirmed our previous finding that rs7730971 was significantly associated with the inclusion of a 158 bp cryptic exon located between exons 9 and 10 (Figure 2). While significant colocalization was not detected using Moloc, we previously found colocalization in this locus using eCaviar.[28] The G allele, which is associated with increased risk of COPD, is also associated with increased inclusion of the cryptic exon (Figure 2B). SpliceAI[29] indicates that rs7730971-G is predicted to slightly increase the splice acceptor strength of a splice site 217 bp upstream of the variant, which corresponds to the 5′ splice acceptor of the cryptic exon, confirming that this is the likely causal variant. Using long-read sequencing, we identified one isoform meeting expression thresholds, which includes the 158 bp cryptic exon (Figure 2C). This transcript includes a premature stop codon in the cryptic exon, and because this stop is more than 50 bases from the transcriptional stop, it would likely be subject to nonsense-mediated decay.[30] This isoform is more abundant in the GG genotype compared to AA (5 reads vs. 1). Supporting the finding that the transcript containing the cryptic exon is subject to nonsense-mediated decay, we found that rs7730971 is also an eQTL for *FBXO38*, with the G allele associated with decreased expression (Figure 2D). We additionally identified a new colocalization between genetic variants in the *FBXO38/HTR4* region and inclusion of an exon at GRCh38 chr5:148415241–148415387 (supplemental information).

## Colocalization analysis of a genetic signal at BTC

Another significant colocalization between sQTLs and COPD GWAS findings was identified in LTRC lung tissue at the *BTC* locus, where we found evidence of a shared genetic signal between variants associated with alternative inclusion of exon 4 of *BTC*, and COPD risk GWAS data (PPA = 0.95) (Figure 3A). The lead colocalized variant was rs62316278 (GRCh38 chr4:74748514C>T), and the COPD risk allele (C) is associated with increased inclusion

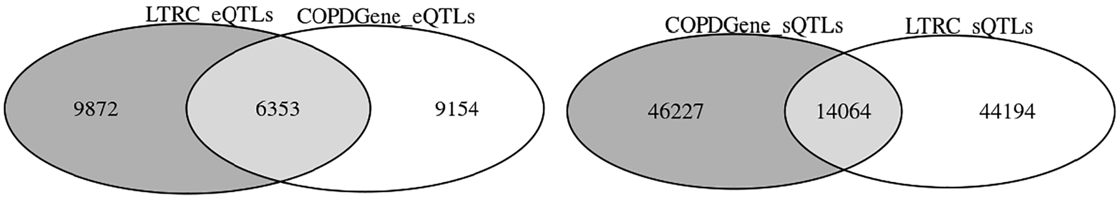


**Figure 1. Overlap between LTRC lung and COPDGene blood eQTLs and sQTLs that were significant at an FDR < 0.05**

of exon 4 (Figure 3B). This variant is within the 95% credible set for sQTL data using SuSie fine mapping, along with 42 additional variants. Of the variants in the 95% credible set, we found using SpliceAI that rs11938093-T (GRCh38 chr4:74750631A>T) is associated with the gain of a splice donor 58 bp upstream of the SNP, corresponding to the splice donor of exon 4, as well as the gain of a splice acceptor 88 bp downstream of the SNP, corresponding to the splice acceptor of exon 4. Long-read sequencing identified four high-abundance isoforms for *BTC* (representing at least 10% of BTC expression each) (Figure 3C), and the proportion of isoforms containing exon 4 was higher in CC vs. TT subjects (84% vs. 48%, $p = 0.0003$). These isoforms correspond to GenBank: NM_001729 (BTC-201, ENST00000395743.8) and NM_001316963 (not included in ENSEMBL). GenBank: NM_001729 (including exon 4) is the primary version of BTC and codes for a 178 amino acid protein, while GenBank: NM_001316963 codes for 129 amino acids.

## Discussion

In this study, we build upon our previous work characterizing COPD-associated sQTLs in blood RNA-seq data from COPDGene by generating a large dataset of eQTLs and sQTLs in human blood and lung tissue and identifying gene expression and splicing events, and we identify a substantial number of QTLs that suggest a functional mechanism for COPD GWAS loci. We found that approximately 50% of splice sites identified were not currently annotated, indicating the vast amount of currently uncharacterized splicing variability present in the transcriptome. Among the 223,128 splice sites identified in LTRC lung tissue and the 160,658 splice sites identified in COPDGene blood, we found that 58,258 (26%) and 60,291 (38%) splice sites, respectively, were associated with at least one variant within 1 Mb (FDR < 5%). In addition, of the 16,266 genes expressed in LTRC and 15,507 genes expressed in COPDGene, 12,225 (75%) and 15,279 (99%) of genes, respectively, were associated with at least one SNP. The majority of eQTL and sQTL SNPs were located upstream of the gene body or in intronic regions, suggesting that many sQTLs function through long-range or indirect effects, as opposed to modifying splice donors or acceptors directly. Alternatively, the identified SNPs may be tagging another SNP in LD, which more directly modifies a splice site. We identified 38 loci (corresponding to 33 of the original 82 GWAS loci) with significant colocalization with either eQTL, sQTL, or apaQTL data, and of these, 9 loci colocalized most strongly with LTRC sQTLs and 15 with COPDGene sQTLs. We confirmed our previous sQTL findings in the *FBXO38/HTR4* region and identified *BTC* as additional target with strong COPD colocalization.

Here, we validated in lung tissue our previous findings from blood that rs7730971 is associated with splicing of a cryptic exon in *FBXO38*. Open reading frame analysis of the full length transcript sequence indicates that the cryptic exon contains a premature stop codon, and

**Table 4. Annotation of QTL variants in relation to the gene body**

| | COPDGene | | LTRC | |
|---|---|---|---|---|
| Annotation | eQTLs (%) | sQTLs (%) | eQTLs (%) | sQTLs (%) |
| Upstream gene variant | 32.1 | 30.1 | 31.8 | 29.4 |
| Intron variant | 24.4 | 28.8 | 25.2 | 25.8 |
| Downstream gene variant | 14.5 | 14.9 | 14.9 | 15.8 |
| Intergenic region | 11.9 | 9.7 | 10.6 | 11.4 |
| 3′ UTR variant | 5.0 | 3.4 | 5.7 | 3.3 |
| 5′ UTR variant | 3.1 | 2.4 | 3.6 | 2.2 |
| Missense variant | 2.1 | 2.5 | 2.2 | 3.3 |
| Synonymous variant | 1.9 | 2.3 | 1.8 | 3.2 |
| Other | 4.9 | 5.8 | 2.8 | 4.2 |

**Table 5. Summary of colocalization analysis**

| | | Lead QTL | | | | | GWAS | |
|---|---|---|---|---|---|---|---|---|
| Best colocalized SNP[a] | PPA[b] | Gene[b] | QTL type | Splice site | p value[d] | Effect[c] | p value | Effect[c] |
| chr4:144567946A>G | 0.973 | *HHIP* | LTRC_sQTL | chr4:144734889–144737129 | 0.00610 | 0.104 | 4.09E−59 | 16.136 |
| chr4:105897896G>A | 0.999 | *NPNT* | LTRC_sQTL | chr4:105927428–105931515 | 8.12E−10 | 0.114 | 3.04E−46 | 14.331 |
| chr5:148475407C>T | 0.973 | *FBXO38* | LTRC_sQTL | chr5:148414306–148415928 | 0.00333 | −0.195 | 2.58E−33 | −12.010 |
| chr15:71329185G>A | 0.992 | *THSD4* | LTRC_eQTL | ENSG00000187720.14 | 0.154 | 4.653 | 1.58E−32 | 11.852 |
| chr16:75439564G>A | 0.933 | *TMEM170A* | COPDGene_sQTL | chr16:75451839–75464232 | 7.63E−08 | 0.187 | 1.26E−20 | 9.277 |
| chr6:30814428G>C | 0.987 | *IER3* | COPDGene_sQTL | chr6:30744196–30744279 | 0.000119 | −0.176 | 1.41E−20 | 9.295 |
| chr4:88948181T>C | 0.832 | *PKD2* | COPDGene_sQTL | chr4:88019571–88035071 | 3.76E−08 | −0.518 | 4.23E−18 | −8.722 |
| chr6:32660630T>C | 0.997 | *HLA-DRB5* | COPDGene_sQTL | chr6:32520345–32555661 | 6.09E−15 | −0.275 | 5.56E−18 | −8.661 |
| chr5:157505976A>T | 0.968 | *ADAM19* | LTRC_eQTL | ENSG00000135074.15 | 8.62E−10 | 2.468 | 1.22E−16 | −8.324 |
| chr3:128242335T>A | 0.935 | *EEFSEC* | COPDGene_sQTL | chr3:128195329–128246836 | 0.0014 | 0.032 | 3.53E−15 | 7.897 |
| chr6:29639324T>C | 0.997 | *HLA-E* | COPDGene_sQTL | chr6:30490515–30491137 | 4.82E−301 | −0.890 | 4.35E−15 | −7.864 |
| chr6:27420975T>C | 0.976 | *ZKSCAN4* | COPDGene_sQTL | chr6:28249834–28251867 | 5.35E−20 | 0.308 | 3.58E−14 | −7.573 |
| chr6:28651576A>G | 0.996 | *GABBR1* | COPDGene_eQTL | ENSG00000204681.11 | 3.96E−17 | −2.034 | 3.82E−14 | −7.556 |
| chr6:26409662G>C | 0.905 | *BTN2A1* | LTRC_sQTL | chr6:26459828–26463244 | 1.79E−10 | −0.486 | 1.03E−13 | 7.449 |
| chr6:32775967G>A | 0.982 | *HLA-DRB5* | COPDGene_sQTL | chr6:32481801–32525584 | 8.02E−09 | −0.222 | 3.76E−12 | 6.955 |
| chr2:238965524G>A | 0.941 | *TWIST2* | LTRC_eQTL | ENSG00000233608.4 | 0.0483 | 0.269 | 2.17E−11 | −6.699 |
| chr3:25496178G>A | 0.990 | *RARB* | LTRC_eQTL | ENSG00000077092.19 | 2.60E−06 | 0.925 | 5.84E−11 | −6.570 |
| chr2:9145396G>A | 0.956 | *LINC00299* | COPDGene_sQTL | chr2:8299793–8312726 | 0.0479 | −0.115 | 2.41E−10 | 6.343 |
| chr1:16979534C>A | 0.990 | *CROCC* | COPDGene_sQTL | chr1:16922798–16924325 | 0.000528 | −0.121 | 6.55E−10 | 6.192 |
| chr7:100032719C>T | 0.994 | *ZSCAN21* | COPDGene_sQTL | chr7:100049841–100051478 | 6.28E−05 | −0.153 | 7.26E−10 | −6.175 |
| chr19:45790878G>A | 0.998 | *DMWD* | LTRC_APAQTL | ENST00000597053.1 | 0.00118 | −0.044 | 1.67E−09 | −6.009 |
| chr1:39582337G>A | 0.933 | *PPIEL* | COPDGene_sQTL | chr1:39548951–39554349 | 0.0176 | −0.128 | 1.98E−09 | 6.009 |
| chr12:95843792T>C | 0.972 | *NTN4* | LTRC_sQTL | chr12:95713338–95717545 | 6.56E−09 | −0.400 | 2.64E−09 | −5.935 |
| chr4:74748514C>T | 0.951 | *BTC* | LTRC_sQTL | chr4:74748149–74750573 | 0.00187 | −0.238 | 3.45E−09 | −5.903 |
| chr1:111195294T>C | 0.850 | *DENND2D* | COPDGene_sQTL | chr1:111194726–111195668 | 0.00843 | −0.556 | 3.59E−09 | −5.892 |
| chr14:92649065G>C | 0.963 | *RIN3* | LTRC_APAQTL | ENST00000553992.1 | 0.00115 | −0.025 | 6.69E−09 | 5.795 |
| chr1:239689643G>C | 0.968 | *CHRM3-AS2* | COPDGene_APAQTL | ENST00000593855 | 2.77E−48 | −0.279 | 9.61E−09 | 5.755 |
| chr11:13145018T>C | 0.941 | *RASSF10* | LTRC_eQTL | ENSG00000189431.7 | 4.27E−09 | 0.250 | 1.18E−08 | 5.717 |
| chr5:151215512A>G | 0.994 | *GM2A* | LTRC_APAQTL | ENST00000523004.1 | 9.43E−100 | 0.011 | 1.40E−08 | −5.695 |
| chr7:2830820T>G | 0.873 | *GNA12* | LTRC_sQTL | chr7:2733501–2762829 | 0.00185 | 0.156 | 1.74E−08 | 5.648 |
| chr17:38730575G>A | 0.990 | *CISD3* | LTRC_eQTL | ENSG00000277972.1 | 8.70E−11 | −2.872 | 1.90E−08 | 5.620 |
| chr3:29430921G>C | 0.985 | *RBMS3* | LTRC_eQTL | ENSG00000144642.21 | 1.02E−11 | −2.128 | 2.04E−08 | 5.636 |
| chr15:49578340A>G | 0.943 | *FAM227B* | LTRC_sQTL | chr15:49589745–49606156 | 0.0146 | −0.151 | 2.82E−08 | 5.564 |
| chr16:58063696G>C | 0.890 | *MMP15* | LTRC_sQTL | chr16:58040698–58041617 | 0.0122 | 0.274 | 4.26E−08 | 5.500 |
| chr10:80458350G>C | 0.995 | *TSPAN14* | COPDGene_sQTL | chr10:80459507–80463088 | 3.64E−07 | 0.132 | 4.30E−08 | −5.465 |
| chr1:45543641G>C | 0.959 | *TESK2* | COPDGene_APAQTL | ENST00000486676 | 0.00967 | −0.032 | 4.67E−08 | −5.485 |
| chr2:42206107C>T | 0.980 | *COX7A2L* | COPDGene_APAQTL | ENST00000463055 | N/A | N/A | 4.85E−08 | −5.446 |
| chr17:30129740C>T | 0.865 | *NSRP1* | COPDGene_sQTL | chr17:30118173–30156218 | 0.000150 | 0.116 | 4.86E−08 | 5.480 |

[a]SNP: chromosome, position, reference, and effect allele, where position is build 38.
[b]PPA: posterior probability of association from Moloc software. For some loci, there were multiple colocalizations between QTLs and GWAS data; the results for the association with the highest PPA are reported here.
[c]The direction of the effect size is based on the second allele listed in the variant ID.
[d]*p* value is for the QTL shown in the "QTL type" column.

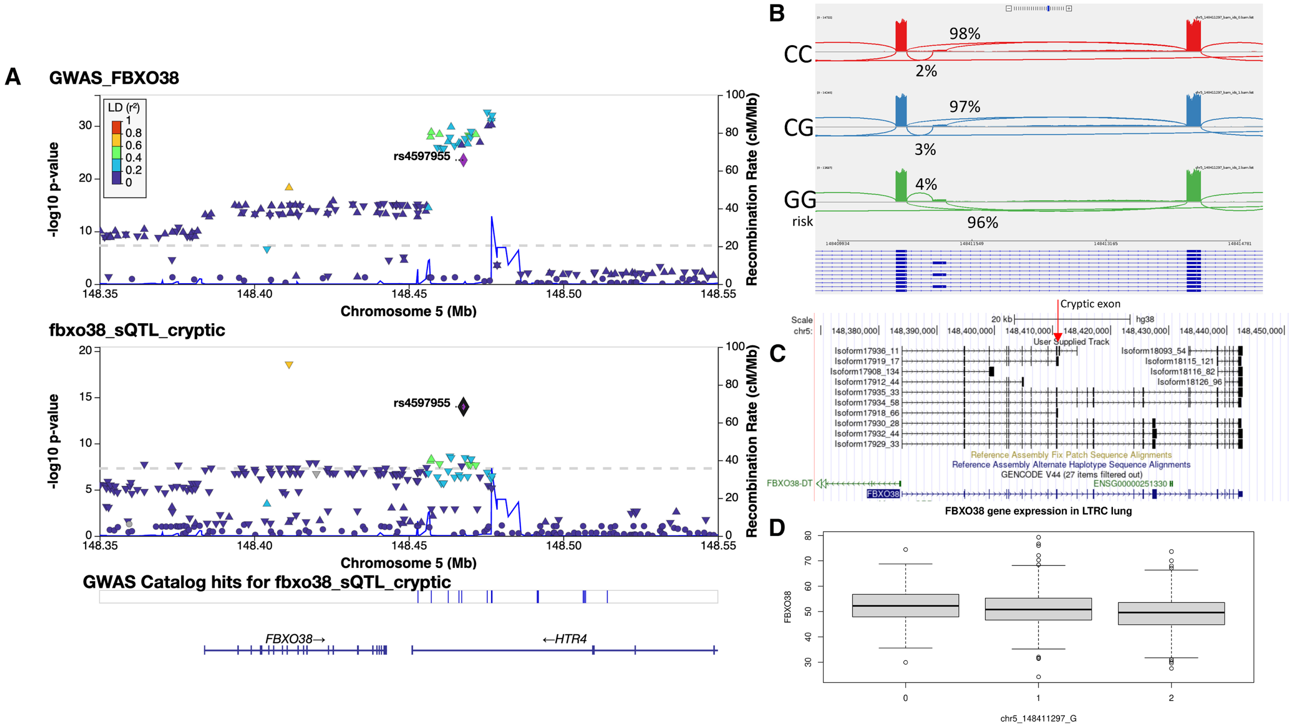


**Figure 2. Replication of FBXO38 sQTL findings in lung tissue**

(A) Locus association plot for COPD GWAS and *FBXO38* sQTLs. The lead SNP associated with FBXO38 splicing, rs7730971, is highlighted in purple and used as the LD reference.

(B) IGV sashimi plot showing the region spanning chr5:148409934–148414781 from lung tissue RNA-seq from 191 subjects from each genotype of rs7730971.

(C) Long-read sequencing data showing all FBXO38 isoforms representing at least 1% of total FBXO38 expression. The cryptic exon is identified with a red arrow.

(D) Boxplot of total gene expression values for *FBXO38*.

therefore, this transcript is likely subject to nonsense-mediated decay. We also found that rs7730971 is associated with gene expression of *FBXO38*, with decreased expression with the G allele, which is also the variant associated with a bioinformatically predicted increase in nonsense-mediated decay. Therefore, the likely mechanism underlying the eQTL association is degradation of *FBXO38* as result of increased inclusion of a cryptic exon, which results in a transcript with an early stop codon. This is an example of a mechanism by which an eQTL can be mediated through an sQTL. While the difference in inclusion of cryptic exon is small, 2% vs. 4% inclusion in the CC vs. GG genotype, this measurement is likely skewed, as the transcript that contains the cryptic exon would be degraded, resulting in decreased quantification of exon inclusion. In other words, the inclusion of the exon in the GG genotype is likely inaccurate due to the degradation of the isoform that contains the exon. Future directions include inhibition of nonsense mediation followed by RNA-seq to quantify the true levels of exon inclusion.

The allele associated with decreased expression of *FBXO38* is associated with increased COPD risk, suggesting that *FBXO38* plays a protective role against COPD. We found an additional colocalization between rs100 37493, the most significant COPD GWAS SNP in the locus, and an additional *FBXO38* exon (shown in the supplemental results). The long-read sequences containing this exon correspond to two predicted isoforms, each encoding shorter proteins than the most abundant isoform. The COPD risk allele is associated with the shortened isoforms, indicating that the full-length *FBXO38* isoform is protective. These shortened isoforms lack the F box domain, which is a component of all members of F box proteins, including FBXO38. F box proteins are a component of the ubiquitin ligase complex and also function as transcription factors.[31] FBXO38, specifically, is a coactivator of the Kruppel-like factor 7 (KLF7) zinc-finger transcription factor.[32,33] While little is known about the function of FBXO38 and its potential role in COPD, the lack of the F box protein, which is responsible for protein-protein interactions, in disease-associated isoforms indicates that FBXO38 interactions are critical for protection against COPD.

We identified an additional colocalization between the COPD GWAS and a previously unidentified candidate gene, *BTC*. We specifically found that rs62316278, the lead GWAS SNP in the *BTC* region, is also associated with splicing of exon 4, with the risk allele (C) increasing exon 4 inclusion. The primary form of *BTC*, GenBank: NM_001729, includes exon 4, and rs62316278-C is

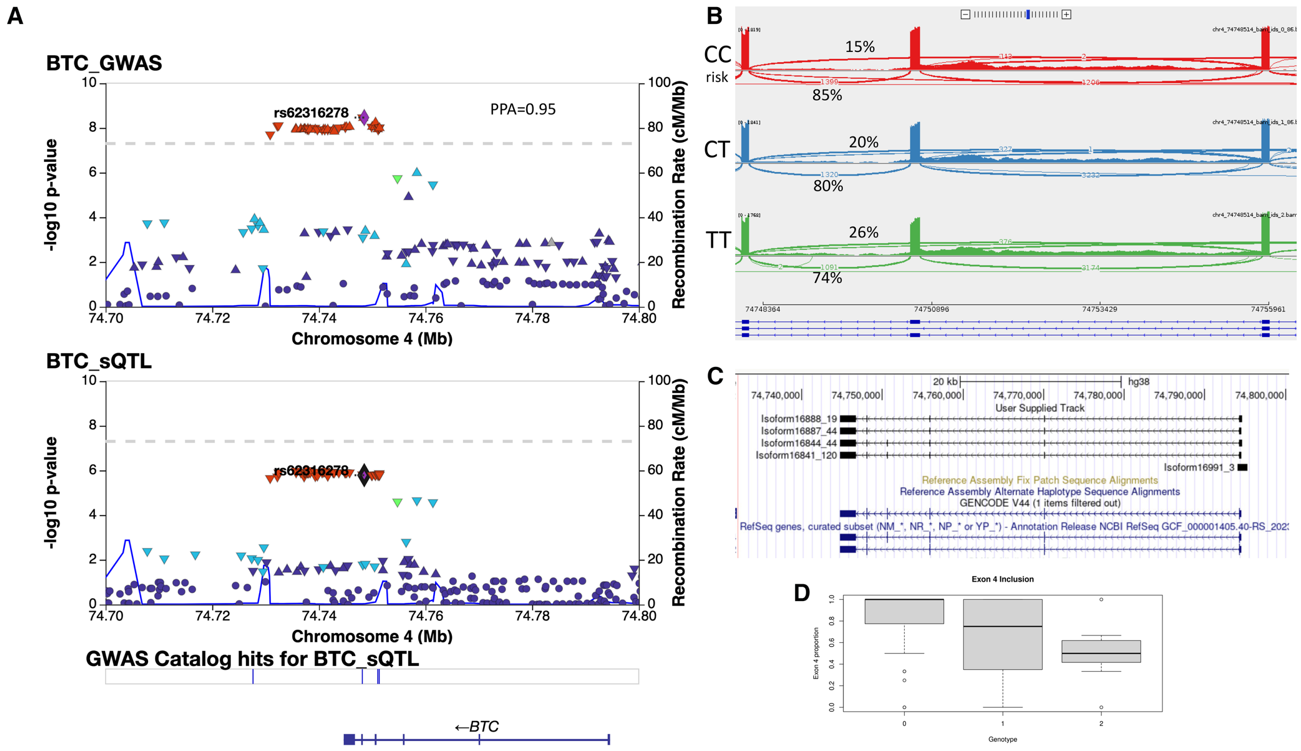


**Figure 3. sQTLs for BTC colocalize with GWAS data for COPD**

(A) Locus association plot for COPD GWAS and *BTC* sQTL. The lead colocalized SNP, rs62316278, is highlighted in purple and used as the LD reference.

(B) IGV sashimi plot showing the region spanning chr4:74748364–74755961 from lung tissue RNA-seq from 86 subjects from each genotype of rs62316278.

(C) Long-read sequencing data showing all BTC isoforms representing at least 10% of total BTC expression.

(D) Proportion of BTC expression of isoforms containing exon 4 by copies of rs62316278-T.

associated with an increased proportion of GenBank: NM_001729 relative to GenBank: NM_001316963, a protein-coding isoform that lacks exon 4. This suggests that reduced inclusion of exon 4 is protective for COPD. BTC is a member of the epidermal growth factor (EGF) family of peptide ligands and a ligand for EGF receptor (EGFR). Human *BTC* encodes a 178 amino acid product corresponding to the BTC precursor protein (pro-BTC) and contains several domains including a signal peptide, an EGF motif, and transmembrane domains.[34] The mature sequence of BTC is cleaved from the extracellular domain of BTC to produce an 80 amino acid protein. Based on the structure of other members of the EGF family, exon 4 is predicted to make up the third loop of the EGF-like motif and the transmembrane domain.[34] The EGF domain is critical to binding with EGF ligands, and therefore, the isoform lacking exon 4 could be predicted to have altered function. Several previous studies have linked *BTC* expression to COPD, and *BTC* has been found to be higher in ex-smokers with COPD than those without COPD, has been associated with emphysema in alpha-1 antitrypsin deficiency,[35] and has been found to be elevated in acute exacerbations in COPD.[36] More targeted work is needed to investigate the function of *BTC* isoforms in COPD.

The major strength of this study is the large sample size, which allowed us to comprehensively characterize alternative splicing in whole blood and lung tissue. Our sample size significantly exceeds that of other commonly used resources, such as the GTEx, which includes 515 lung samples and 670 blood samples. While we sought colocalization with Global Initiative for Chronic Obstructive Disease (GOLD)-defined COPD, we expect that other respiratory- or smoking-related genetic associations would benefit from the use of this resource. One potential weakness is that our RNA-seq was performed using whole-lung tissue and whole-blood samples, which contain a variety of cell types. Therefore, some of the changes in transcriptional splicing detected may actually be reflecting differences in inter-individual cell proportions. Another limitation is the use of Moloc, which attempts colocalization with the most significant signal in the region and may not be optimal in the setting of multiple QTL or GWAS signals in the region. In addition, many GWAS loci colocalized with multiple QTL types and with multiple genes. While we characterized the colocalization with the highest PPA for each locus, it is possible that some loci can be explained by multiple genes and mechanisms. A full list of all colocalizations with all QTL types is shown in Table S2. Comparisons of the number of QTLs or the number of colocalizations between lung tissue and blood tissue may be influenced by the different library preparation methodology used for COPDGene blood compared to

LTRC lung RNA-seq data. In COPDGene, total RNA was sequenced, which allows for the inclusion of transient and incompletely processed RNA, while poly(A) selection was used in LTRC. Additional work characterizing splicing using single-cell short- or long-read RNA-seq is required.

In conclusion, we discovered that multiple COPD GWAS associations colocalize with sQTLs and identified or replicated several candidate genes as COPD targets for follow-up.


## Acknowledgments

This work was funded by NIH grants K01HL157613, R01HL157879, P01HL114501, X01HL139404, R01HL124233, R01HL126596, R01HL153248, R01HL149861, R01 HL111527HL135142, and NIGMS R35 GM140844. Molecular data for the Trans-Omics in Precision Medicine (TOPMed) program were supported by the National Heart, Lung, and Blood Institute (NHLBI). Whole-genome sequencing and RNA-seq for "NHLBI TOPMed: The Lung Tissue Research Consortium (phs001662)" was performed at the Northwest Genome Center (NWGC; HHSN268201600032I, RNA-seq), and broad genomics (HHSN268201600034I, WGS) core support, including centralized genomic read mapping and genotype calling, along with variant quality metrics and filtering, were provided by the TOPMed Informatics Research Center (3R01HL-117626-02S1; contract HHSN268201800002I). Core support including phenotype harmonization, data management, sample-identity QC, and general program coordination were provided by the TOPMed Data Coordinating Center (R01HL-120393 and U01HL-120393; contract HHSN268201800001I). We gratefully acknowledge the studies and participants who provided biological samples and data for TOPMed. The COPDGene study (NCT00608764) is supported by grants from the NHLBI (U01HL089897 and U01HL089856), by NIH contract 75N92023D00011, and by the COPD Foundation through contributions made to an industry advisory committee that has included AstraZeneca, Bayer Pharmaceuticals, Boehringer-Ingelheim, Genentech, GlaxoSmithKline, Novartis, Pfizer, and Sunovion. This study utilized biological specimens and data provided by the Lung Tissue Research Consortium (LTRC) supported by the NHLBI.


## Author contributions

Conceptualization, A.S., P.J.C., and C.P.H.; data curation, M.H.C., P.J.C., C.P.H., C.V., and E.K.S.; formal analysis, A.S., W.K., and R.P.C.; writing – original draft preparation, A.S. and C.P.H.; writing – review & editing, A.S., W.K., R.P.C., C.V., E.K.S., M.H.C., P.J.C., and C.P.H.

## Declaration of interests

P.J.C. received grant support from Bayer and Sanofi and consulting fees from Verona Pharmaceuticals. C.P.H. has received research grants from Bayer, Boehringer-Ingelheim, and Vertex and consulting fees from Chiesi, Sanofi, and Takeda. M.H.C. has received grant funding from Bayer. In the past 3 years, E.K.S. received grant support from Bayer and Northpond Laboratories.

## Supplemental information

Supplemental information can be found online at https://doi.org/10.1016/j.xhgg.2025.100493.

## Web resources

GenBank, https://www.ncbi.nlm.nih.gov/genbank/


Received: January 29, 2025
Accepted: August 6, 2025


## References


1. Cohen, B.H., Ball, W.C., Jr., Brashears, S., Diamond, E.L., Kreiss, P., Levy, D.A., Menkes, H.A., Permutt, S., and Tockman, M.S. (1977). Risk factors in chronic obstructive pulmonary disease (COPD). Am. J. Epidemiol. *105*, 223–232. https://doi.org/10.1093/oxfordjournals.aje.a112378.
2. Kueppers, F., Miller, R.D., Gordon, H., Hepper, N.G., and Offord, K. (1977). Familial prevalence of chronic obstructive pulmonary disease in a matched pair study. Am. J. Med. *63*, 336–342. https://doi.org/10.1016/0002-9343(77)90270-4.
3. McCloskey, S.C., Patel, B.D., Hinchliffe, S.J., Reid, E.D., Wareham, N.J., and Lomas, D.A. (2001). Siblings of patients with severe chronic obstructive pulmonary disease have a significant risk of airflow obstruction. Am. J. Respir. Crit. Care Med. *164*, 1419–1424. https://doi.org/10.1164/ajrccm.164.8.2105002.
4. Silverman, E.K., Chapman, H.A., Drazen, J.M., Weiss, S.T., Rosner, B., Campbell, E.J., O'DONNELL, W.J., Reilly, J.J., Ginns, L., Mentzer, S., et al. (1998). Genetic epidemiology of severe, early-onset chronic obstructive pulmonary disease. Risk to relatives for airflow obstruction and chronic bronchitis. Am. J. Respir. Crit. Care Med. *157*, 1770–1778. https://doi.org/10.1164/ajrccm.157.6.9706014.
5. Sakornsakolpat, P., Prokopenko, D., Lamontagne, M., Reeve, N.F., Guyatt, A.L., Jackson, V.E., Shrine, N., Qiao, D., Bartz, T. M., Kim, D.K., et al. (2019). Genetic landscape of chronic obstructive pulmonary disease identifies heterogeneous cell-type and phenotype associations. Nat. Genet. *51*, 494–505. https://doi.org/10.1038/s41588-018-0342-2.
6. Hobbs, B.D., de Jong, K., Lamontagne, M., Bossé, Y., Shrine, N., Artigas, M.S., Wain, L.V., Hall, I.P., Jackson, V.E., Wyss, A.B., et al. (2017). Genetic loci associated with chronic obstructive pulmonary disease overlap with loci for lung function and pulmonary fibrosis. Nat. Genet. *49*, 426–432. https://doi.org/10.1038/ng.3752.
7. Shrine, N., Guyatt, A.L., Erzurumluoglu, A.M., Jackson, V.E., Hobbs, B.D., Melbourne, C.A., Batini, C., Fawcett, K.A., Song, K., Sakornsakolpat, P., et al. (2019). New genetic signals for lung function highlight pathways and chronic obstructive pulmonary disease associations across multiple ancestries. Nat. Genet. *51*, 481–493. https://doi.org/10.1038/s41588-018-0321-7.
8. Shrine, N., Izquierdo, A.G., Chen, J., Packer, R., Hall, R.J., Guyatt, A.L., Batini, C., Thompson, R.J., Pavuluri, C., Malik, V., et al. (2023). Multi-ancestry genome-wide association analyses improve resolution of genes and pathways influencing lung function and chronic obstructive pulmonary disease risk. Nat. Genet. *55*, 410–422. https://doi.org/10.1038/s41588-023-01314-0.

9. Gamazon, E.R., Segrè, A.V., van de Bunt, M., Wen, X., Xi, H. S., Hormozdiari, F., Ongen, H., Konkashbaev, A., Derks, E.M., Aguet, F., et al. (2018). Using an atlas of gene regulation across 44 human tissues to inform complex disease- and trait-associated variation. Nat. Genet. *50*, 956–967. https://doi.org/10.1038/s41588-018-0154-4.
10. Saferali, A., Yun, J.H., Parker, M.M., Sakornsakolpat, P., Chase, R.P., Lamb, A., Hobbs, B.D., Boezen, M.H., Dai, X., de Jong, K., et al. (2019). Analysis of genetically driven alternative splicing identifies FBXO38 as a novel COPD susceptibility gene. PLoS Genet. *15*, e1008229. https://doi.org/10.1371/journal.pgen.1008229.
11. Barbeira, A.N., Dickinson, S.P., Bonazzola, R., Zheng, J., Wheeler, H.E., Torres, J.M., Torstenson, E.S., Shah, K.P., Garcia, T., Edwards, T.L., et al. (2018). Exploring the phenotypic consequences of tissue specific gene expression variation inferred from GWAS summary statistics. Nat. Commun. *9*, 1825. https://doi.org/10.1038/s41467-018-03621-1.
12. Regan, E.A., Hokanson, J.E., Murphy, J.R., Make, B., Lynch, D.A., Beaty, T.H., Curran-Everett, D., Silverman, E.K., and Crapo, J.D. (2010). Genetic epidemiology of COPD (COPDGene) study design. COPD J. Chronic Obstr. Pulm. Dis. *7*, 32–43. https://doi.org/10.3109/15412550903499522.
13. Ghosh, A.J., Hobbs, B.D., Yun, J.H., Saferali, A., Moll, M., Xu, Z., Chase, R.P., Morrow, J., Ziniti, J., Sciurba, F., et al. (2022). Lung tissue shows divergent gene expression between chronic obstructive pulmonary disease and idiopathic pulmonary fibrosis. Respir. Res. *23*, 97. https://doi.org/10.1186/s12931-022-02013-w.
14. Parker, M.M., Chase, R.P., Lamb, A., Reyes, A., Saferali, A., Yun, J.H., Himes, B.E., Silverman, E.K., Hersh, C.P., and Castaldi, P.J. (2017). RNA sequencing identifies novel non-coding RNA and exon-specific effects associated with cigarette smoking. BMC Med. Genom. *10*, 58.
15. Patro, R., Duggal, G., Love, M.I., Irizarry, R.A., and Kingsford, C. (2017). Salmon provides fast and bias-aware quantification of transcript expression. Nat. Methods *14*, 417–419. https://doi.org/10.1038/nmeth.4197.
16. Soneson, C., Love, M.I., and Robinson, M.D. (2015). Differential analyses for RNA-seq: transcript-level estimates improve gene-level inferences. F1000Res. *4*, 1521. https://doi.org/10.12688/f1000research.7563.2.
17. Li, Y.I., Knowles, D.A., Humphrey, J., Barbeira, A.N., Dickinson, S.P., Im, H.K., and Pritchard, J.K. (2018). Annotation-free quantification of RNA splicing using LeafCutter. Nat. Genet. *50*, 151–158. https://doi.org/10.1038/s41588-017-0004-9.
18. Risso, D., Ngai, J., Speed, T.P., and Dudoit, S. (2014). Normalization of RNA-seq data using factor analysis of control genes or samples. Nat. Biotechnol. *32*, 896–902. https://doi.org/10.1038/nbt.2931.
19. Taylor-Weiner, A., Aguet, F., Haradhvala, N.J., Gosai, S., Anand, S., Kim, J., Ardlie, K., Van Allen, E.M., and Getz, G. (2019). Scaling computational genomics to millions of individuals with GPUs. Genome Biol. *20*, 228. https://doi.org/10.1186/s13059-019-1836-7.
20. Cho, M.H., Castaldi, P.J., Wan, E.S., Siedlinski, M., Hersh, C. P., Demeo, D.L., Himes, B.E., Sylvia, J.S., Klanderman, B.J., Ziniti, J.P., et al. (2012). A genome-wide association study of COPD identifies a susceptibility locus on chromosome 19q13. Hum. Mol. Genet. *21*, 947–957. https://doi.org/10.1093/hmg/ddr524.
21. Wang, K., Li, M., and Hakonarson, H. (2010). ANNOVAR: functional annotation of genetic variants from high-throughput sequencing data. Nucleic Acids Res. *38*, e164. https://doi.org/10.1093/nar/gkq603.
22. GTEx Consortium (2020). The GTEx Consortium atlas of genetic regulatory effects across human tissues. Science *369*, 1318–1330. https://doi.org/10.1126/science.aaz1776.
23. Giambartolomei, C., Zhenli Liu, J., Zhang, W., Hauberg, M., Shi, H., Boocock, J., Pickrell, J., Jaffe, A.E., CommonMind Consortium, Pasaniuc, B., and Roussos, P. (2018). A Bayesian framework for multiple trait colocalization from summary association statistics. Bioinformatics *34*, 2538–2545. https://doi.org/10.1093/bioinformatics/bty147.
24. Zou, Y., Carbonetto, P., Xie, D., Wang, G., and Stephens, M. (2024). Fast and flexible joint fine-mapping of multiple traits via the Sum of Single Effects model. Preprint at bioRxiv. https://doi.org/10.1101/2023.04.14.536893.
25. Saferali, A., Kim, W., Xu, Z., Chase, R.P., Cho, M.H., Laederach, A., Castaldi, P.J., and Hersh, C.P. (2024). Colocalization analysis of 3' UTR alternative polyadenylation quantitative trait loci reveals novel mechanisms underlying associations with lung function. Hum. Mol. Genet. *33*, 1164–1175. https://doi.org/10.1093/hmg/ddae055.
26. Saferali, A., Xu, Z., Sheynkman, G.M., Hersh, C.P., Cho, M. H., Silverman, E.K., Laederach, A., Vollmers, C., and Castaldi, P.J. (2020). Characterization of a COPD-Associated NPNT Functional Splicing Genetic Variant in Human Lung Tissue via Long-Read Sequencing. Preprint at medRxiv. https://doi.org/10.1101/2020.10.20.20203927.
27. Morrow, J.D., Zhou, X., Lao, T., Jiang, Z., Demeo, D.L., Cho, M.H., Qiu, W., Cloonan, S., Pinto-Plata, V., Celli, B., et al. (2017). Functional interactors of three genome-wide association study genes are differentially expressed in severe chronic obstructive pulmonary disease lung tissue. Sci. Rep. *7*, 44232.
28. Hormozdiari, F., van de Bunt, M., Segrè, A.V., Li, X., Joo, J.W. J., Bilow, M., Sul, J.H., Sankararaman, S., Pasaniuc, B., Eskin, E., et al. (2016). Colocalization of GWAS and eQTL Signals Detects Target Genes. Am. J. Hum. Genet. *99*, 1245 ,Äì60-,Äì60.
29. Jaganathan, K., Kyriazopoulou Panagiotopoulou, S., McRae, J.F., Darbandi, S.F., Knowles, D., Li, Y.I., Kosmicki, J.A., Arbelaez, J., Cui, W., Schwartz, G.B., et al. (2019). Predicting Splicing from Primary Sequence with Deep Learning. Cell *176*, 535–548.e24. https://doi.org/10.1016/j.cell.2018.12.015.
30. Zhang, Z., Xin, D., Wang, P., Zhou, L., Hu, L., Kong, X., and Hurst, L.D. (2009). Noisy splicing, more than expression regulation, explains why some exons are subject to nonsense-mediated mRNA decay. BMC Biol. *7*, 23. https://doi.org/10.1186/1741-7007-7-23.
31. Kipreos, E.T., and Pagano, M. (2000). The F-box protein family. Genome Biol. *1*, REVIEWS3002. https://doi.org/10.1186/gb-2000-1-5-reviews3002.
32. Smaldone, S., Laub, F., Else, C., Dragomir, C., and Ramirez, F. (2004). Identification of MoKA, a novel F-box protein that modulates Kruppel-like transcription factor 7 activity. Mol. Cell Biol. *24*, 1058–1069. https://doi.org/10.1128/MCB.24.3.1058-1069.2004.
33. Smaldone, S., and Ramirez, F. (2006). Multiple pathways regulate intracellular shuttling of MoKA, a co-activator of

transcription factor KLF7. Nucleic Acids Res. *34*, 5060–5068. https://doi.org/10.1093/nar/gkl659.

34. Dunbar, A.J., and Goddard, C. (2000). Structure-function and biological role of betacellulin. Int. J. Biochem. Cell Biol. *32*, 805–815. https://doi.org/10.1016/s1357-2725(00)00028-5.
35. Serban, K.A., Pratte, K.A., Strange, C., Sandhaus, R.A., Turner, A.M., Beiko, T., Spittle, D.A., Maier, L., Hamzeh, N., Silverman, E.K., et al. (2022). Unique and shared systemic biomarkers for emphysema in Alpha-1 Antitrypsin deficiency and chronic obstructive pulmonary disease. EBioMedicine *84*, 104262. https://doi.org/10.1016/j.ebiom.2022.104262.
36. Chen, H., Song, Z., Qian, M., Bai, C., and Wang, X. (2012). Selection of disease-specific biomarkers by integrating inflammatory mediators with clinical informatics in AECOPD patients: a preliminary study. J. Cell Mol. Med. *16*, 1286–1297. https://doi.org/10.1111/j.1582-4934.2011.01416.x.

**HGGA, Volume 7**

## Supplemental information

# Overlap between COPD genetic association results and transcriptional quantitative trait loci

**Aabida Saferali, Wonji Kim, Robert P. Chase, NHLBI TransOmics in Precision Medicine (TOPMed), Christopher Vollmers, Edwin K. Silverman, Michael H. Cho, Peter J. Castaldi, and Craig P. Hersh**

**<u>Supplementary Results</u>:**

*<u>Identification of an additional colocalization between FBXO38 splicing and COPD GWAS</u>*

We found that rs10037493-T (5:148475407:C:T), which is associated with increased COPD risk is also associated with decreased inclusion of the identified exon (Supplementary Figure 1 a-b). This variant has a 71% probability of being causal for the sQTL association according to SuSie finemapping [23]. While long read sequencing did not identify a confirmed isoform containing this exon (ie in the isoforms passing filtering thresholds), in the raw RNAseq reads we identified at least 15 reads supporting the presence of this exon (Supplementary Figure 1c and Supplementary Figure 2). These reads correspond to two predicted protein-coding isoforms, XM_017009902 and XM_011537684, which would code for 713 and 788 amino acid proteins which differ from the usual 1188 amino acid protein

**Supplementary Figures**

Supplementary Figure 1

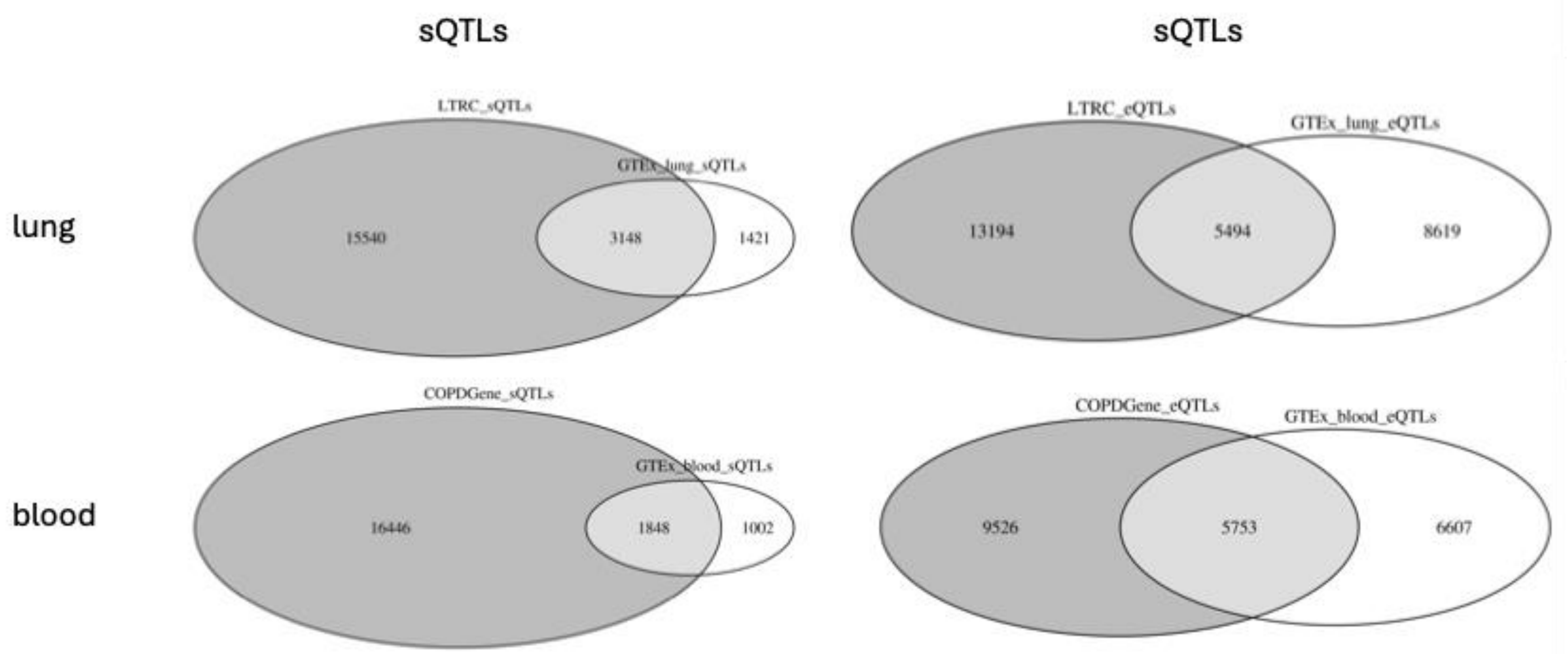


**Supplementary Figure 1:** Overlap between LTRC and COPDGene eQTLs and sQTLs with GTEx eQTLs and sQTL from lung tissue and blood. sQTL results are shown for splice clusters (instead of individual introns/junctions).

Supplementary Figure 2

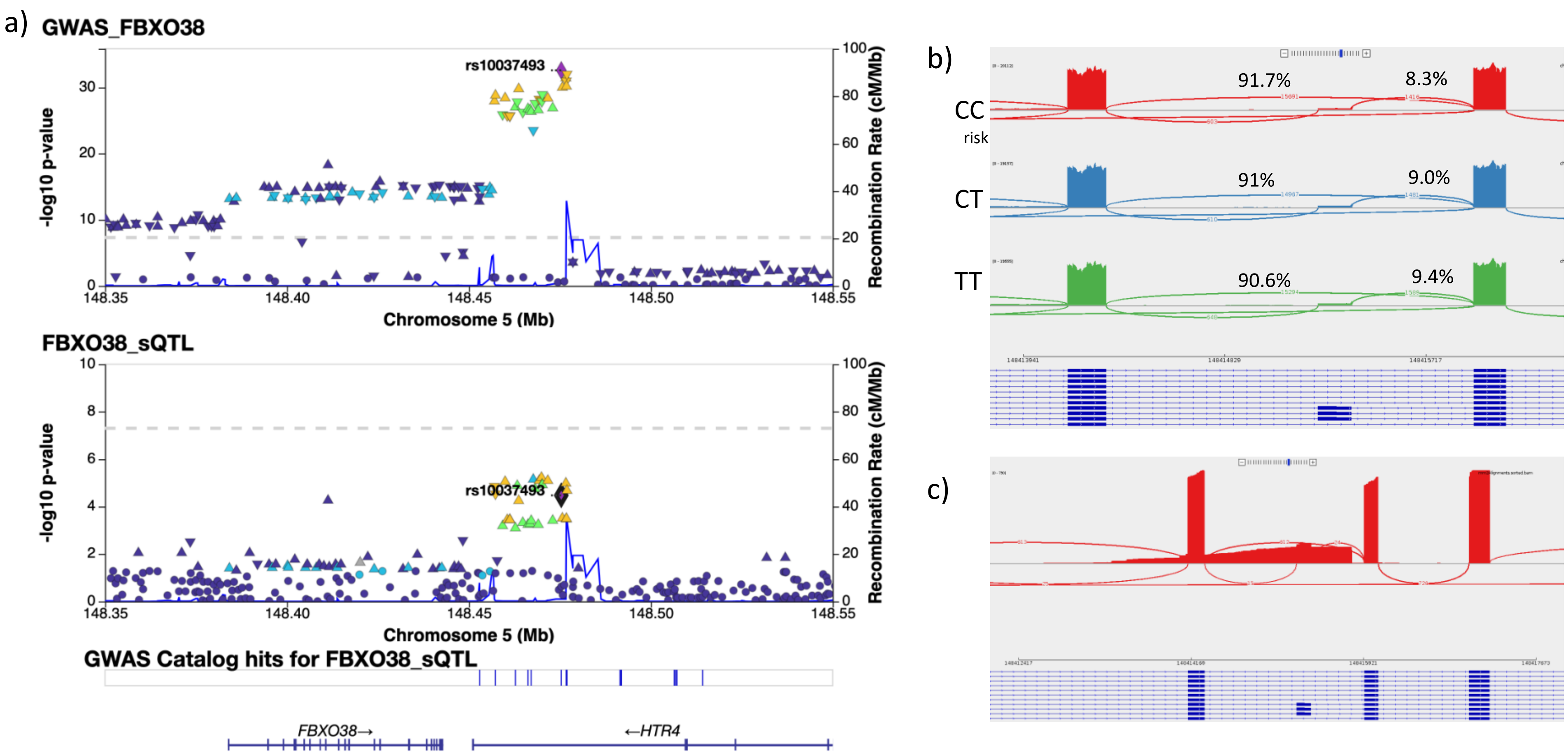


**Supplementary Figure 2: An additional colocalization between *FBXO38* sQTLs and COPD.** a) Locus association plot for COPD GWAS and *FBXO38* sQTL. The lead colocalized SNP, rs10037493, is highlighted in purple and used as the LD reference. b) IGV sashimi plot showing the region spanning chr5:148413941-148416605 for 273 subjects from each genotype of rs10037493. c) Long read sequencing data showing raw FBXO38 reads of isoforms containing the exon of interest.

Supplementary Figure 3

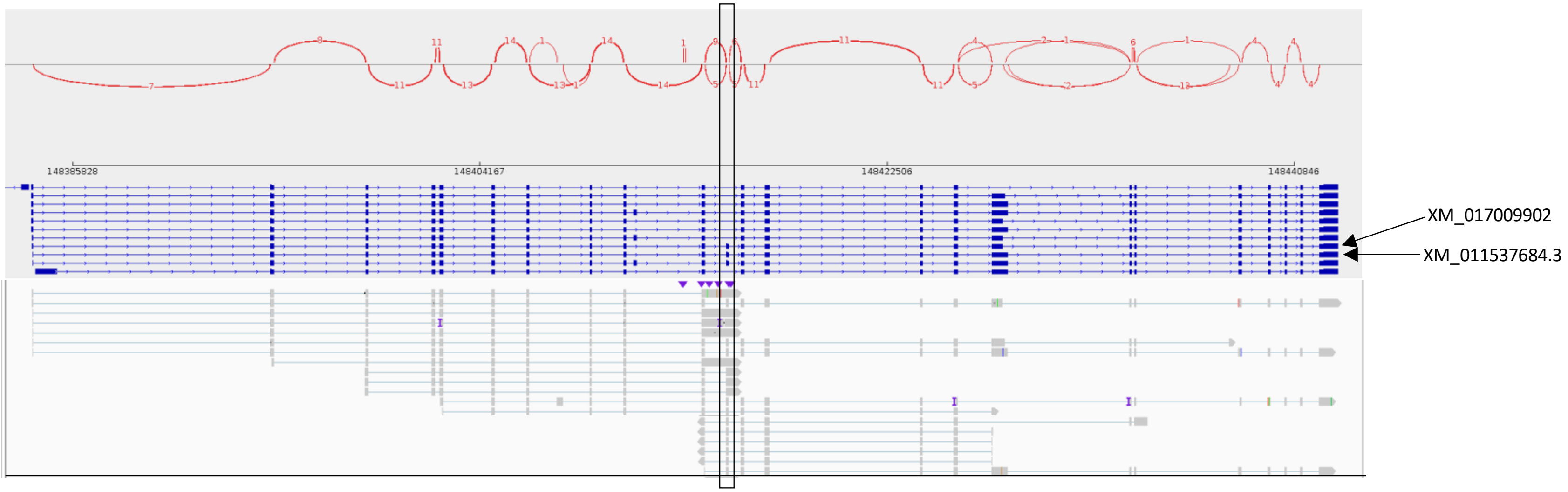


**Supplementary Figure3: IGV plot of raw long read sequencing data from FBXO38 showing reads containing the exon**

## Supplementary Table 1: 82 COPD GWAS loci identified in previous studies

| lead GWAS variant | lead variant rsID | window variant | Colocalization window start | colocalization window end | Lead variant effect allele | Lead variant beta | Lead variant p |
|---|---|---|---|---|---|---|---|
| 1:16979534:C:A | rs9435731 | 1:16979534:C:A | 15979534 | 17979534 | A | 0.0613 | 6.55E-10 |
| 1:39594353:G:A | rs76841360 | 1:39594353:G:A | 38594353 | 40594353 | A | 0.0728 | 4.95E-10 |
| 1:45480964:G:T | rs4660861 | 1:45480964:G:T | 44480964 | 46480964 | T | -0.0549 | 4.42E-08 |
| 1:60447471:C:T | rs72673419 | 1:60447471:C:T | 59447471 | 61447471 | T | 0.1299 | 4.00E-09 |
| 1:111195486:C:T | rs629619 | 1:111195486:C:T | 110195486 | 112195486 | T | 0.078 | 2.94E-10 |
| 1:218515813:T:C | rs3009947 | 1:218515813:T:C | 217515813 | 219515813 | T | -0.0567 | 4.02E-09 |
| 1:219751552:T:A | rs11118406 | 1:219751552:T:A | 218751552 | 220751552 | A | -0.0725 | 4.11E-11 |
| 1:239737706:G:C | rs11579382 | 1:239737706:G:C | 238737706 | 240737706 | C | 0.0582 | 6.50E-09 |
| 2:9150228:C:T | rs955277 | 2:9150228:C:T | 8150228 | 10150228 | T | 0.0652 | 1.90E-10 |
| 2:15766055:C:T | rs10929386 | 2:15766055:C:T | 14766055 | 16766055 | T | -0.0573 | 9.11E-09 |
| 2:42206107:C:T | rs12466981 | 2:42206107:C:T | 41206107 | 43206107 | T | -0.061 | 4.85E-08 |
| 2:156156523:C:T | rs72902175 | 2:156156523:C:T | 155156523 | 157156523 | T | 0.0824 | 1.63E-08 |
| 2:217818431:A:G | rs2571445 | 2:217818431:A:G | 216818431 | 218818431 | A | 0.0701 | 3.43E-12 |
| 2:228705203:C:G | rs16825267 | 2:228705203:C:G | 227705203 | 229705203 | C | 0.1762 | 1.75E-20 |
| 2:238951008:C:T | rs62191105 | 2:238951008:C:T | 237951008 | 239951008 | T | -0.089 | 2.58E-12 |
| 3:11599127:G:A | rs2442776 | 3:11599127:G:A | 10599127 | 12599127 | A | -0.0879 | 1.99E-10 |
| 3:25479091:C:A | rs1529672 | 3:25479091:C:A | 24479091 | 26479091 | A | -0.0867 | 2.54E-11 |
| 3:29430921:G:C | rs13073544 | 3:29430921:G:C | 28430921 | 30430921 | C | 0.062 | 2.04E-08 |
| 3:55124197:A:G | rs17759204 | 3:55124197:A:G | 54124197 | 56124197 | A | -0.0713 | 8.81E-11 |
| 3:57760788:C:T | rs62259026 | 3:57760788:C:T | 56760788 | 58760788 | T | -0.0647 | 2.44E-08 |
| 3:123358195:T:A | rs4093840 | 3:123358195:T:A | 122358195 | 124358195 | A | 0.0621 | 3.91E-10 |
| 3:128242335:T:A | rs2955083 | 3:128242335:T:A | 127242335 | 129242335 | A | 0.1224 | 3.53E-15 |
| 3:141428572:T:C | rs7650602 | 3:141428572:T:C | 140428572 | 142428572 | T | -0.0546 | 4.86E-08 |
| 3:169028357:G:A | rs7642001 | 3:169028357:G:A | 168028357 | 170028357 | A | 0.078 | 1.08E-14 |
| 4:74748153:G:A | rs4585380 | 4:74748153:G:A | 73748153 | 75748153 | A | -0.0665 | 3.40E-09 |
| 4:88962667:G:A | rs7671261 | 4:88962667:G:A | 87962667 | 89962667 | A | 0.0855 | 1.43E-18 |
| 4:105897896:G:A | rs34712979 | 4:105897896:G:A | 104897896 | 106897896 | A | 0.1691 | 3.04E-46 |
| 4:144567946:A:G | rs13140176 | 4:144567946:A:G | 143567946 | 145567946 | A | 0.1662 | 4.09E-59 |
| 5:52899203:G:A | rs1551943 | 5:52899203:G:A | 51899203 | 53899203 | A | 0.0725 | 5.83E-10 |
| 5:72848178:C:T | rs34651 | 5:72848178:C:T | 71848178 | 73848178 | T | -0.1012 | 3.02E-08 |
| 5:95700996:C:T | rs153916 | 5:95700996:C:T | 94700996 | 96700996 | T | 0.0602 | 6.32E-10 |
| 5:133103318:T:A | rs62375246 | 5:133103318:T:A | 132103318 | 134103318 | A | 0.0626 | 2.22E-08 |
| 5:148475407:C:T | rs10037493 | 5:148475407:C:T | 147475407 | 149475407 | T | -0.1189 | 2.58E-33 |
| 5:151215512:A:G | rs979453 | 5:151215512:A:G | 150215512 | 152215512 | A | -0.0598 | 1.40E-08 |
| 5:157510035:A:G | rs10866659 | 5:157510035:A:G | 156510035 | 158510035 | A | -0.0849 | 1.17E-16 |
| 5:171474582:A:T | rs12519165 | 5:171474582:A:T | 170474582 | 172474582 | A | 0.0653 | 1.06E-09 |

| | | | | | | | |
|---|---|---|---|---|---|---|---|
| 6:7211585:G:A | rs1334576 | 6:7211585:G:A | 6211585 | 8211585 | A | 0.0555 | 1.19E-08 |
| 6:19842430:C:T | rs9350191 | 6:19842430:C:T | 18842430 | 20842430 | T | 0.1113 | 5.11E-14 |
| 6:22004680:C:T | rs13198656 | 6:22004680:C:T<br>6:25417423:G:T<br>6:26509382:C:T<br>6:27669976:A:G<br>6:28712247:A:G | 21004680<br>24417423<br>25509382<br>26669976<br>27712247 | 23004680<br>26417423<br>27509382<br>28669976<br>29712247 | T | 0.0608 | 1.23E-09 |
| 6:30745803:T:C | rs2284174 | 6:30745803:T:C | 29745803 | 31745803 | T | -0.1174 | 2.10E-21 |
| 6:32183666:C:T | rs2070600 | 6:32183666:C:T<br>6:32628407:T:C<br>6:33710967:C:A | 31183666<br>31628407<br>32710967 | 33183666<br>33628407<br>34710967 | T | -0.1865 | 1.09E-17 |
| 6:108945052:T:C | rs2806356 | 6:108945052:T:C | 107945052 | 109945052 | T | -0.0977 | 2.89E-15 |
| 6:116935855:T:C | rs674621 | 6:116935855:T:C | 115935855 | 117935855 | T | -0.0603 | 7.61E-09 |
| 6:139959261:T:C | rs646695 | 6:139959261:T:C | 138959261 | 140959261 | T | -0.0747 | 4.62E-11 |
| 6:142347764:T:C | rs9399401 | 6:142347764:T:C | 141347764 | 143347764 | T | 0.147 | 1.64E-40 |
| 7:2712518:G:A | rs798565 | 7:2712518:G:A | 1712518 | 3712518 | A | -0.0654 | 3.93E-09 |
| 7:20378511:C:T | rs2040732 | 7:20378511:C:T | 19378511 | 21378511 | T | -0.0581 | 6.89E-09 |
| 7:100032719:C:T | rs2897075 | 7:100032719:C:T | 99032719 | 101032719 | T | -0.0636 | 7.26E-10 |
| 8:8840148:C:G | rs9329170 | 8:8840148:C:G | 7840148 | 9840148 | C | 0.0924 | 3.56E-10 |
| 9:4143749:A:T | rs10114763 | 9:4143749:A:T | 3143749 | 5143749 | A | -0.0711 | 8.66E-13 |
| 9:23588686:T:C | rs156394 | 9:23588686:T:C | 22588686 | 24588686 | T | 0.0702 | 3.79E-12 |
| 9:82511248:T:C | rs7866939 | 9:82511248:T:C | 81511248 | 83511248 | T | -0.0593 | 1.75E-08 |
| 9:98899368:G:A | rs10760580 | 9:98899368:G:A | 97899368 | 99899368 | A | -0.0717 | 1.18E-10 |
| 9:116639371:G:A | rs803923 | 9:116639371:G:A | 115639371 | 117639371 | A | 0.0546 | 2.72E-08 |
| 10:12235993:C:T | rs7068966 | 10:12235993:C:T | 11235993 | 13235993 | T | -0.0959 | 6.22E-23 |
| 10:76559121:A:C | rs2579762 | 10:76559121:A:C | 75559121 | 77559121 | A | -0.0625 | 2.58E-10 |
| 10:79946568:A:G | rs721917 | 10:79946568:A:G | 78946568 | 80946568 | A | -0.0544 | 2.21E-08 |
| 10:103897116:G:A | rs1570221 | 10:103897116:G:A | 102897116 | 104897116 | A | 0.0575 | 2.22E-08 |
| 11:13149689:C:T | rs4757118 | 11:13149689:C:T | 12149689 | 14149689 | T | 0.059 | 3.79E-09 |
| 11:86733719:A:G | rs117261012 | 11:86733719:A:G | 85733719 | 87733719 | A | -0.0837 | 6.91E-10 |
| 12:28167603:T:A | rs11049386 | 12:28167603:T:A | 27167603 | 29167603 | A | -0.0618 | 2.70E-08 |
| 12:95843792:T:C | rs7307510 | 12:95843792:T:C | 94843792 | 96843792 | T | -0.0736 | 2.64E-09 |
| 12:115510096:A:G | rs7958945 | 12:115510096:A:G | 114510096 | 116510096 | A | -0.0627 | 1.03E-09 |
| 13:44268367:G:A | rs9525927 | 13:44268367:G:A | 43268367 | 45268367 | A | -0.0735 | 2.78E-09 |
| 14:92639608:G:C | rs72699855 | 14:92639608:G:C<br>14:94349487:T:C | 91639608<br>93349487 | 93639608<br>95349487 | C | -0.0749 | 4.82E-09 |
| 15:49692513:G:C | rs72731149 | 15:49692513:G:C | 48692513 | 50692513 | C | -0.1114 | 8.27E-09 |
| 15:71320175:T:G | rs1441358 | 15:71320175:T:G | 70320175 | 72320175 | T | -0.1208 | 7.39E-33 |
| 15:78606590:C:G | rs55676755 | 15:78606590:C:G | 77606590 | 79606590 | C | -0.1088 | 2.75E-26 |
| 15:83724155:G:A | rs10152300 | 15:83724155:G:A | 82724155 | 84724155 | A | -0.081 | 4.20E-12 |
| 16:10615156:T:C | rs56134392 | 16:10615156:T:C | 9615156 | 11615156 | T | -0.057 | 4.51E-08 |
| 16:57988721:G:A | rs8044657 | 16:57988721:G:A | 56988721 | 58988721 | A | -0.1032 | 1.12E-08 |
| 16:75306333:A:T | rs4888379 | 16:75306333:A:T | 74306333 | 76306333 | A | -0.0926 | 5.86E-21 |

| | | | | | | | |
|---|---|---|---|---|---|---|---|
| 17:30086111:T:C | rs8080772 | 17:30086111:T:C | 29086111 | 31086111 | T | 0.0593 | 1.37E-08 |
| 17:38678826:C:T | rs34727469 | 17:38678826:C:T | 37678826 | 39678826 | T | 0.0827 | 1.07E-08 |
| 17:40062520:G:A | rs62065216 | 17:40062520:G:A | 39062520 | 41062520 | A | 0.0586 | 8.07E-09 |
| 17:45846834:C:G | rs12373142 | 17:45846834:C:G | 44846834 | 46846834 | C | -0.073 | 9.94E-10 |
| 17:71220546:T:C | rs11655567 | 17:71220546:T:C | 70220546 | 72220546 | T | -0.0588 | 1.90E-09 |
| 18:8808466:T:C | rs647097 | 18:8808466:T:C | 7808466 | 9808466 | T | -0.0742 | 1.03E-11 |
| 19:45790878:G:A | rs72626215 | 19:45790878:G:A | 44790878 | 46790878 | A | -0.0685 | 1.67E-09 |
| 21:34289445:A:C | rs2096468 | 21:34289445:A:C | 33289445 | 35289445 | A | 0.0541 | 4.00E-08 |
| 22:18006117:G:C | rs9617650 | 22:18006117:G:C | 17006117 | 19006117 | C | -0.079 | 4.44E-10 |
| 22:32939401:C:G | rs73158393 | 22:32939401:C:G | 31939401 | 33939401 | C | 0.0666 | 7.67E-09 |

**Supplementary Table 2: Additional colocalization signals**

| Lead GWAS variant | Best colocalized SNP | Locus | Best colocalized Gene | Best Colocalized Type | Other colocalized genes | GWAS identified target gene |
|---|---|---|---|---|---|---|
| 4:144567946:A:G | 4:144567946:A:G | 4q31.21 | HHIP | LTRC_sQTL | LTRC_eQTL_HHIP_0.9 | HHIP |
| 4:105897896:G:A | 4:105897896:G:A | 4q24 | NPNT | LTRC_sQTL | | PPA2, NPNT |
| 5:148475407:C:T | 5:148475407:C:T | 5q32 | FBXO38 | LTRC_sQTL | COPDGene_sQTL_ABLIM3_0.89 | FBXO38 |
| 15:71320175:T:G | 15:71329185:G:A | 15q23 | THSD4 | LTRC_eQTL | LTRC_eQTL_THSD4_0.99 | THSD4, LARP6 |
| 16:75306333:A:T | 16:75439564:G:A | 16q23.1 | TMEM170A | COPDGene_sQTL | COPDGene_sQTL_CFDP1_0.8 | CFDP1, TERF2IP, CTRB2 |
| 6:30745803:T:C | 6:30814428:G:C | 6p21.33 | IER3 | COPDGene_sQTL | LTRC_sQTL_FLOT1_0.97<br>LTRC_sQTL_PPT2-EGFL8_0.85<br>LTRC_sQTL_NRM_0.8 | IER3, FLOT1, TUBB, DDR1, VARS2 ,SFTA2, CDSN |
| 4:88962667:G:A | 4:88948181:T:C | 4q22.1 | PKD2 | COPDGene_sQTL | | FAM13A, NAP1L5,PPM1K |
| 6:32183666:C:T | 6:32660630:T:C | 6p21.32 | . | COPDGene_sQTL | LTRC_eQTL_AGER_0.99<br>LTRC_sQTL_PSORS1C1_0.95<br>COPDGene_sQTL_CCHCR1_0.95<br>COPDGene_eQTL_VARS2_0.94<br>COPDGene_sQTL_VARS2_0.94<br>LTRC_sQTL_NOTCH4_0.93<br>LTRC_sQTL_LST1_0.93<br>LTRC_sQTL_AIF1_0.92<br>COPDGene_sQTL_AGER_0.9<br>LTRC_sQTL_CCHCR1_0.88<br>LTRC_sQTL_SFTA2_0.86<br>LTRC_sQTL_VARS2_0.8 | AGER, NOTCH4, AGPAT1, CYP21A2, MSH5, LY6G6E HLA-DQB1 |
| 5:157510035:A:G | 5:157505976:A:T | 5q33.3 | ADAM19 | LTRC_eQTL | COPDGene_sQTL_ADAM19_0.8<br>9 | ADAM19, CYFIP2, CLINT1, SOX30, C5orf52 |
| 3:128242335:T:A | 3:128242335:T:A | 3q21.3 | EEFSEC | COPDGene_sQTL | COPDGene_sQTL_RUVBL1_0.91 | RUVBL1, GATA2 |
| 2:238951008:C:T | 2:238965524:G:A | 2q37.3 | TWIST2 | LTRC_eQTL | | UBE2F |
| 3:25479091:C:A | 3:25496178:G:A | 3p24.2 | RARB | LTRC_eQTL | | RARB |

| | | | | | | |
|---|---|---|---|---|---|---|
| 2:9150228:C:T | 2:9145396:G:A | 2p25.1 | LINC00299 | COPDGene_sQTL | | ASAP2 |
| 1:16979534:C:A | 1:16979534:C:A | 1p36.13 | CROCC | COPDGene_sQTL | LTRC_sQTL_MST1P2_0.98<br>LTRC_eQTL_ATP13A2_0.98<br>LTRC_sQTL_CROCC_0.98<br>LTRC_sQTL_MFAP2_0.98<br>LTRC_sQTL_CROCCP2_0.97<br>LTRC_eQTL_MFAP2_0.96<br>LTRC_APAQTL_MFAP2_0.82 | MFAP2<br>PADI2 |
| 7:100032719:C:T | 7:100032719:C:T | 7q22.1 | ZSCAN21 | COPDGene_sQTL | COPDGene_eQTL_MCM7_0.98<br>COPDGene_APAQTL_ZKSCAN1_0.97<br>COPDGene_APAQTL_STAG3L5P_0.96<br>COPDGene_sQTL_CNPY4_0.95<br>LTRC_sQTL_COPS6_0.93<br>LTRC_sQTL_ZSCAN21_0.8 | ZKSCAN1, ZNF3, AZGP1, AP4M1, CNPY4, TRIM4, PVRIG, NYAP1 |
| 19:45790878:G:A | 19:45790878:G:A | 19q13.32 | DMWD | LTRC_APAQTL | LTRC_APAQTL_SYMPK_0.97<br>LTRC_sQTL_QPCTL_0.82 | DMPK, SNRPD2 |
| 1:39594353:G:A | 1:39582337:G:A | 1p34.3 | PPIEL | COPDGene_sQTL | COPDGene_sQTL_MACF1_0.88<br>COPDGene_sQTL_PABPC4_0.85 | PABPC4, OXCT2, MYCL |
| 12:95843792:T:C | 12:95843792:T:C | 12q23.1 | NTN4 | LTRC_sQTL | COPDGene_sQTL_CFAP54_0.88 | AMDHD1 |
| 4:74748153:G:A | 4:74748514:C:T | 4q13.3 | BTC | LTRC_sQTL | | EPGN |
| 1:111195486:C:T | 1:111195294:T:C | 1p13.3 | DENND2D | COPDGene_sQTL | LTRC_eQTL_KCNA2_0.84 | DRAM2, OVGP1, INKA2 |
| 14:92639608:G:C | 14:92649065:G:C | 14q32.12 | RIN3 | LTRC_APAQTL | | CPSF2, RIN3, ITPK1 |
| 1:239737706:G:C | 1:239689643:G:C | 1q43 | CHRM3-AS2 | COPDGene APAQTL | COPDGene_sQTL_CHRM3-AS2_0.95 | |
| 11:13149689:C:T | 11:13145018:T:C | 11p15.2 | RASSF10 | LTRC_eQTL | | PARVA |

| | | | | | | |
|---|---|---|---|---|---|---|
| 5:151215512:A:G | 5:151215512:A:G | 5q33.1 | GM2A | LTRC_APAQTL | COPDGene_APAQTL_GM2A_0.95 | |
| 7:2712518:G:A | 7:2830820:T:G | 7p22.3 | GNA12 | LTRC_sQTL | | GNA12 |
| 17:38678826:C:T | 17:38730575:G:A | 17q12 | CISD3 | LTRC_eQTL | LTRC_eQTL_PCGF2_0.94<br>COPDGene_eQTL_MLLT6_0.94<br>LTRC_eQTL_EPOP_0.92<br>COPDGene_sQTL_MLLT6_0.9<br>COPDGene_eQTL_AC006449.6_0.88 | CACNB1 |
| 3:29430921:G:C | 3:29430921:G:C | 3p24.1 | RBMS3 | LTRC_eQTL | | RBMS3 |
| 15:49692513:G:C | 15:49578340:A:G | 15q21.2 | FAM227B | LTRC_sQTL | COPDGene_sQTL_FAM227B_0.92<br>COPDGene_sQTL_AP4E1_0.9 | FAM227B, SLC27A2 |
| 16:57988721:G:A | 16:58063696:G:C | 16q21 | MMP15 | LTRC_sQTL | | CNGB1 |
| 10:79946568:A:G | 10:80458350:G:C | 10q22.3 | TSPAN14 | COPDGene_sQTL | COPDGene_sQTL_FAM213A_0.95<br>LTRC_APAQTL_SFTPA2_0.89<br>LTRC_sQTL_DYDC2_0.89 | TMEM254, DYDC2, FAM213A |
| 1:45480964:G:T | 1:45543641:G:C | 1p34.1 | TESK2 | COPDGene APAQTL | COPDGene_eQTL_AKR1A1_0.95<br>COPDGene_sQTL_AKR1A1_0.95<br>COPDGene_APAQTL_MUTYH_0.93<br>LTRC_sQTL_AKR1A1_0.87 | MUTYH, IPP, UROD |
| 2:42206107:C:T | 2:42206107:C:T | 2p21 | COX7A2L | COPDGene APAQTL | | EML4 |
| 17:30086111:T:C | 17:30129740:C:T | 17q11.2 | NSRP1 | COPDGene_sQTL | LTRC_sQTL_NSRP1_0.82 | SLC6A4, EFCAB5, GOSR1 CORO6 |

**<u>Supplementary Methods</u>: Long Read Sequencing**

*cDNA synthesis*

100 ng of RNA was mixed with dNTP and RNA Inhibitor, then denatured to remove secondary structure for 3 minutes at 72C. Prior to reverse transcription (RT), indexed oligo(dT) primers were added to the mix at 50C. First strand synthesis using Maxima H(-) reverse transcriptase (Thermo Fisher) and a template switching oligo was performed for 90 minutes at 50C. The cDNA was then amplified using PCR with SeqAmp polymerase, ISPCR primer, and Nextera A index primer (95C for 1 minute, 15 cycles of 98C for 15 seconds, 65C for 30 seconds, 68C for 6 minutes, 72C for 7 minutes, 4C hold). Each sample was indexed using a unique combination of olgo(dT) primer and Nextera A index primer. cDNA was cleaned up using ProNex Size Selective Purification System (Promega) at a 1:1.2 ratio.

*Enrichment*

After quantification by Qubit, equal amounts cDNA from every sample were pooled together. 3 ug of cDNA pool were target enriched using the xGEN Hybridization Capture (Integrated DNA Technologies). xGen Human Cot DNA and Blocking Oligos were added to the cDNA pool and cleaned using SPRI beads at a 1.8:1 ratio. The beads were eluted in a mix of xGen 2X Hybridization Buffer, xGen Hybridization Buffer Enhancer, and xGen Hyb Panel and incubated at room temperature for 5 min. The beads were removed using a magnet, and the solution incubated at 95C for 30 seconds, 65C for 4 hours, and a 65C hold. Streptavidin beads were added and incubated at 65C for 45 minutes. The mixture was washed using Wash Buffer 1 and Stringent Wash Buffer at 65C. Then, Wash Buffer 1, Wash Buffer 2, and Wash Buffer 3 were used to wash the mixture at room temperature. PCR was performed on the final solution with SeqAmp polymerase, ISPCR primer, and Nextera A Universal primer for 15 cycles (95C for 1 minute, 98C for 15 seconds, 65C for 30 seconds, 68C for 6 minutes, 72C for 7 minutes, 4C hold). The final enriched library was cleaned up using SPRI beads at 0.65:1 ratio.

*R2C2 library preparation and sequencing*

DNA splints were generated through primer extension as previously described using 2 complementary DNA oligos. Enriched cDNA libraries were circularized by Gibson assembly (NEBbuilder HiFi) with a  DNA splint with ends complementary to the cDNA. ExoI, ExoIII, and Lambda Exonuclease (NEB) were added and incubated for 16 hours at 37C to remove non-circular molecules followed by heat inactivation for 20 minutes at 80C. The reaction was then cleaned using SPRI beads at a 0.8:1 ratio. The clean, circularized library was used as the template for rolling circle amplification (RCA) using Phi29 (NEB) with a random hexamer primer for 18 hours at 30C, and then heat inactivated for 10 minutes at 65C. The reaction was then debranched using T7 endonuclease (NEB) for 2 hours at 37C before being cleaned and concentrated using a Monarch PCR and DNA cleanup kit (NEB).The resulting R2C2 DNA was then run on a 1% agarose gel and DNA  >10kb was excised and purified using a Monarch DNA Gel extraction kit (NEB).

Following the manufacturer's protocol, we then used the Genomic DNA by Ligation (SQK-LS114) kit from ONT to generate sequencing libraries from this size-selected R2C2 DNA. The final library was loaded onto a PromethION R10.4 flow cell and run at 400bp/s. Approximately once per day, flow cells were flushed and treated with DNAse I, then loaded with additional library to increase sequencing throughput.

Resulting raw reads were basecalled using the SUP model of guppy (v6) and consensus called and demultiplexed using C3POa (v2.3).

Oligos used:

**cDNA synthesis**

```
ISPCR primer          AAGCAGTGGTATCAACGCAGAGTAC
Oligo_dT_Index1       AAGCAGTGGTATCAACGCAGAGT [10nt index] ACTTTTTTTTTTTTTTTTTTTTTTTTTTTTTTVN
TSO1_Nextera          TCGTCGGCAGCGTCAGATGTGTATAAGAGArCAG  rUGA  ArU  rUC  TGGTrGrGrG
```

| | |
|---|---|
| Nextera_Primer_A1 | AATGATACGGCGACCACCGAGATCTACAC [8nt index] TCGTCGGCAGCGTCAGATG |

## Enrichment

| | |
|---|---|
| NextA_F_Blocking | AATGATACGGCGACCACCGAGATCTACAC IIIIIIII TCGTCGGCAGCGTCAGATGTGTATAAGAGACAG/3ddC/ |
| NextA_RC_Blocking | CTGTCTCTTATACACATCTGACGCTGCCGACGA IIIIIIII GTGTAGATCTCGGTGGTCGCCGTATCATT |
| RC_OligodT | AA AAA AAA AAA AAA AAA AAA AAA AAA AAA AGT III III III IAC TCT GCG TTG ATA CCA CTG CTT |
| IndexedOligodTBlocker | AAG CAG TGG TAT CAA CGC AGA GTI III III III ACT TTT TTT TTT TTT TTT TTT TTT TTT TTT TT/3ddC/ |

## Splint Synthesis

| | |
|---|---|
| Nextera_A_Splint_1_F | GATCTCGGTGGTCGCCGTATCATT TGAGGCTGATGAGTTCCATA NNNNNTATATNNNNN ATCACTACTTAGTTTTTGATAGCTTCAAGCCAGAGTTGTCTTTTTCTCTTTGCTGGCAGTAAAAG |
| ISPCR_Splint_1_R | ACTCTGCGTTGATACCACTGCTT AAAGGGATATTTTCGATCGC NNNNNATATANNNNN TTAGTGCATTTGATCCTTTTACTCCTCCTAAAGAACAACCTGACCCAGCAAAAGGTACACAATACTTTTACTGCCAGCAAAGAG |
| Nextera_A_Splint_2_F | GATCTCGGTGGTCGCCGTATCATT TGCCGGTTGGGTATCAATAA NNNNNTATATNNNNN ATTGCCTTTATTCTATCTACTTAGTTTTGGCGATGTAGTCTACCTATCCTGATGCTGAATAAAGGC |
| ISPCR_Splint_2_R | ACTCTGCGTTGATACCACTGCTT AATTAGGTTCTAGGATCACG NNNNNATATANNNNN CTGCCATCGAAAATTTTTCACCCGTAACAAGAACTTACAACTCTCTGACGCCTATATCATGAAGGCCTTTATTCAGCATCAGGA |
| Nextera_A_Splint_3_F | GATCTCGGTGGTCGCCGTATCATT GTCGTGATCAAACATTGGGT NNNNNTATATNNNNN TAAAAGTTTTCTGTGTCCATTACGTTTTTTGGAGACGGTCTCAACTATTCTTAATCTCGGCGAACT |
| ISPCR_Splint_3_R | ACTCTGCGTTGATACCACTGCTT AAGTGAGTCGTATTACAGCT NNNNNATATANNNNN ATATTCAGTTCGAAACCTTACCTCCTCTGAAAAGTGTCCCCGAAATCTCTAGGAAATTAACCCAAGTTCGCCGAGATTAAGAAT |
| Nextera_A_Splint_4_F | GATCTCGGTGGTCGCCGTATCATT TAGTGTAAGGTAGCATCCGT NNNNNTATATNNNNN TACGATCATATTATGTTCGTCTGTCTTTAACGGGCTATACTTCTTCTTTAGGAAAGGTCTGAACCT |
| ISPCR_Splint_4_R | ACTCTGCGTTGATACCACTGCTT CTACTATTTGAGTAAAGGCG NNNNNATATANNNNN ACCTGTGTATTAGACCCCACTACTACCCTTAAGATTCCATAACCTTAAGTAGAACCCAGATTGAAGGTTCAGACCTTTCCTAAA |
